# Abundance profiling of specific gene groups using precomputed gut metagenomes yields novel biological hypotheses

**DOI:** 10.1371/journal.pone.0176154

**Published:** 2017-04-27

**Authors:** Konstantin Yarygin, Alexander Tyakht, Andrey Larin, Elena Kostryukova, Sergei Kolchenko, Vilgelm Bitner, Dmitry Alexeev

**Affiliations:** 1 Federal Research and Clinical Centre of Physical-Chemical Medicine (FRCC CPM), Malaya Pirogovskaya 1a, Moscow 119435, Russia; 2 Moscow Institute of Physics and Technology, Institutsky lane 9, Dolgoprudny, Moscow Region, 141700, Russia; 3 HPC HUB LLC, Prospect Mira 112, Bld.12, Moscow 129626, Russia; University of Illinois at Urbana-Champaign, UNITED STATES

## Abstract

The gut microbiota is essentially a multifunctional bioreactor within a human being. The exploration of its enormous metabolic potential provides insights into the mechanisms underlying microbial ecology and interactions with the host. The data obtained using “shotgun” metagenomics capture information about the whole spectrum of microbial functions. However, each new study presenting new sequencing data tends to extract only a little of the information concerning the metabolic potential and often omits specific functions. A meta-analysis of the available data with an emphasis on biomedically relevant gene groups can unveil new global trends in the gut microbiota. As a step toward the reuse of metagenomic data, we developed a method for the quantitative profiling of user-defined groups of genes in human gut metagenomes. This method is based on the quick analysis of a gene coverage matrix obtained by pre-mapping the metagenomic reads to a global gut microbial catalogue. The method was applied to profile the abundance of several gene groups related to antibiotic resistance, phages, biosynthesis clusters and carbohydrate degradation in 784 metagenomes from healthy populations worldwide and patients with inflammatory bowel diseases and obesity. We discovered country-wise functional specifics in gut resistome and virome compositions. The most distinct features of the disease microbiota were found for Crohn’s disease, followed by ulcerative colitis and obesity. Profiling of the genes belonging to crAssphage showed that its abundance varied across the world populations and was not associated with clinical status. We demonstrated temporal resilience of crAssphage and the influence of the sample preparation protocol on its detected abundance. Our approach offers a convenient method to add value to accumulated “shotgun” metagenomic data by helping researchers state and assess novel biological hypotheses.

## Introduction

The human gut microbiota forms a complex ecosystem consisting of hundreds of bacterial species [1]. It is a metabolically active “organ”, with its total metabolic potential covering most reactions known to occur in living organisms. Its gene repertoire has been elucidated in a number of large-scale metagenomic studies, revealing millions of genes that are two orders of magnitude higher than the genes of its host [2]. As the gut community has lived in tight long-term coexistence with the host, many of the identified functions confer various benefits to humans. For instance, gut bacteria are able to ferment indigestible dietary polysaccharides to produce short-chain fatty acids [3], metabolize xenobiotics [4] and human-produced metabolites (i.e., bile acids [5]), synthesize vitamins and other beneficial substances [6] [7] and provide protection from pathogens [8]. Functional analysis of the microbiota encompasses a family of approaches for the qualitative and quantitative profiling of specific gene sets involved in each of these functions.

A number of experimental approaches have been applied for the functional analysis of specific gut microbial genes. One of these approaches is functional screening (functional metagenomics) in which the constructed metagenomic DNA library is screened for a specific activity. Examples of its application include profiling of enzymes involved in carbohydrate [3] and xenobiotic metabolism [9], which has implications for diet and medicine. Another method exploits bioreactors that physically model the environment and the structure of the human intestinal tract. One benefit is freedom from most of the ethical issues raised during work with human subjects. Thus, five-stage reactors that model the small intestine and colon have been developed [10]. Here, the focus of the examination is the enzymatic activity of bacteria inhabiting the reactor under various concentrations of nutrients (i.e., polysaccharides) and other parameters.

The rise of high-throughput DNA sequencing has provided unprecedented insight into the functions of the human gut microbiota based on its metagenome. In one of its formats (amplicon sequencing), the genes of interest are amplified using specific primers and sequenced to provide a portrait of the genetic diversity in the sample. Although the majority of amplicon surveys target the 16S rRNA marker gene for taxonomic rather than functional analysis, the method has also been applied to various microbiotas for the analysis of genes related to drug resistance [11] and bile acid metabolism [12]. Although a large number of sequence reads provide high depth to the analysis, the drawback is that only one or a few genes can be assessed at a time. Whole-genome (“shotgun”) metagenomic sequencing generates genomic reads of the total genetic material of all community members and thus provides a rich data source for functional metagenomic analysis and assessment of the total metabolic potential of the community. With tens of millions of short metagenomic reads per metagenome available, one common approach to obtain a functional portrait is based on prior *de novo* assembly and mapping of the reads to contigs. For example, as a part of the MetaHIT project, metagenomic reads from 124 gut metagenomes were assembled *de novo* to obtain a representative 3.3 mln gut microbial gene catalogue [2]] a useful reference for the analysis of new metagenomic datasets. The genes can be pooled by groups [i.e., COG (Clusters of Orthologous Groups) or KEGG (Kyoto Encyclopedia of Genes and Genomes) orthology] and pathways that can then be compared between groups to yield differentially abundant features. However, assembly is not only computationally but also memory-demanding. An alternative method is based on aligning the reads to a representative reference gene/genome set and normalizing the coverage depth to obtain relative abundance values for individual genes. Software packages for functional analysis of metagenomes like HUMAnN [13] or MEGAN [14] are used to align metagenomic reads against gene sequences from KEGG database and calculate abundances to identify the pathways present in the metagenome. This method requires an order of magnitude lower memory volume but still considerable computational resources.

Although it is possible to analyze the whole spectrum of functions simultaneously, there are a number of gene groups that are traditionally of particular interest during gut microbiota functional analyses. Some of these groups are the enzymes responsible for carbohydrate metabolism, including the synthesis or degradation of complex and simple sugars. Dietary fiber, which is a major component abundant in vegetables and grains, cannot be digested by humans and is metabolized by the microbiota. The essential products of fermentation are short-chain fatty acids (SCFA), mainly acetate, butyrate and propionate. SCFAs not only provide 10% of the daily caloric intake but also play an important role in gut homeostasis and immunity regulation [15] [16]. Thus, the relative abundance of the carbohydrate degradation genes is potentially an important biomarker to be considered for studies of the microbiota in health and disease.

Another clinically important group of genes is the antibiotic resistance (AR) genes. The wide application of antibiotics in medicine and agriculture has resulted in the global spread of AR genes in microbes, including pathogens, which represents a major public health problem. Gut microbes can transfer AR genes with the help of mobile genetic elements both within the gut and with microbes from other environments (i.e., the soil [17] or animal microbiota [18]). Thus, the human gut microbiota is considered an important reservoir of the resistome. Obviously, AR genes can be identified in the microbiota using functional selection [19]; however, “shotgun” metagenomic sequencing is a more robust method for AR genes identification because, unlike functional metagenomics, it does not require expression of all the AR genes in the surrogate host [20]. Recent large-scale studies of world populations identified a rich country-specific diversity of AR genes in gut metagenomes [21] [22].

The list of the newly examined specific activities of the gut microbiota and the studies highlighting the genes responsible for these functions keeps growing. Some of these activities include xenobiotic degradation [4], butyrate production [23], biosynthesis of small molecules [7] and bacteriophage genes [24]. Here, we developed an effective method for the qualitative profiling of a user-defined gene group in gut metagenomic data based on the pre-alignment of the metagenomic data against a global reference gene catalogue. We demonstrate the applicability of our method by estimating the relative abundance of several groups of clinically relevant genes, including antibiotic resistance, carbohydrate degradation, biosynthesis, and phage taxonomic markers, in metagenomic datasets from recently published international large-scale studies. The analysis revealed interesting inter- and intra-country variations in the gene group levels. The validity of our approach was confirmed by a number of alternative bioinformatic methods. Our approach is less memory-demanding than the methods using de novo assembly and more computationally efficient than direct mapping to a reference due to reuse of precomputed gene abundance levels. Our approach provides a computationally efficient and flexible method to estimate the facets of the functional potential of the human gut microbiota, which is a biomedically important object.

## Materials and methods

### Workflow of the method

The workflow of our method for calculating the relative abundance of user-defined gene groups in metagenomes contains two basic steps. Firstly, we calculate the abundance for each of the 3.3 mln genes represented in MetaHIT gene catalogue [2] in each metagenome (this step is performed only once; the obtained data are then re-used for any user-defined gene group). Next, the correspondence between the sequences in gene catalogue and sequences of gene group of interest is determined.

Initially, the reads of each metagenome are aligned against a non-redundant catalogue containing 3.3 mln sequences from gut microbial genes. The mapping is performed with Bowtie v0.12.8 [25] using the following parameters: -t -f -v 3 -k 1. The key -C was added for SOLiD color-space reads. The relative abundance values for each gene were calculated by normalizing total length of successfully mapped reads by gene length and the total number of reads in the metagenome:

$$\begin{array}{c} Gene\;relative\;abundance=\frac{\sum_{Successfully\;mapped\;reads}Read\;length}{Gene\;length*Total\;number\;of\;reads\;in\;the\;metagenome} \end{array}$$

For each specific gene group of interest, its sequences are aligned against the gene catalogue using BLASTn or tBLASTn (in cases where the gene group is represented by nucleotide or amino acid sequences, respectively) with the following similarity criteria: e-value < 1e-5, percent identity > 80%, alignment length/query length > 0.8, and alignment length/subject length > 0.8. The search might return multiple hits per gene. To obtain the profile of the gene group for each metagenome, the relative abundance of each gene obtained during the pre-mapping step is extracted from the database and summed across the respective hits. In cases when multiple query genes are aligned to the same sequence from the catalogue, the abundance of that sequence is assigned to each gene in the group. This causes unavoidable “spreading” of the abundance values among homologous genes. Increasing stringency of similarity criteria allows to increase the method precision, but reduces its sensitivity (see S1 File for precision/recall values for different similarity criteria). We have selected a balanced option that results in 10% recall and 82% precision. Low recall is mainly due to the low-abundant genes (S1 Fig)—thus only slightly impacting the overall relative abundance of genes and subsequent analysis.

The database with the precomputed catalogue gene abundance levels and other data required for processing user metagenomes and gene groups is bundled with the source code as text files. The script *add_new_metagenomes.py* allows the user to process his or her new metagenomic datasets by mapping them to the global gene catalogue and recording the gene level information into the database. The script *get_genes_abund.py* calculates the relative abundance table for a user-defined gene group (in FASTA format) in the selected metagenomic datasets. Both scripts are available on GitHub: https://github.com/KonstantinYarygin/Yarygin_2016. We recommend that scripts be run on a parallel computing cluster satisfying the basic requirements of the Bowtie software. Mapping of an average read set (22 mln reads) to the catalogue requires < 5.0 Gb of RAM. To reduce the BLAST and Bowtie execution times, we recommend running them in multiple threads (here *n* = 20 was used). The analysis was performed on the FRCC PCM computer cluster [cluster with 12 worker nodes (ten nodes with 2x Opteron 6176 with 12 cores with 64 Gb of RAM, two nodes with 4x Opteron 6176 with 12 cores of 2.3 GHz with 256 Gb of RAM, and one master node) and the HPC HUB LLC cloud infrastructure [cluster with four worker nodes (14 vCPU on physical 2×8 cores Xeon E5-2650 v2, 128 Gb of RAM) and one master node].

### Gut metagenomic datasets and gene groups

All analyses were performed for 781 published gut metagenomes belonging to healthy Danish [26], American [27], Chinese [28] and Russian [29] populations, as well as obese Danish subjects [26] and Spanish patients with ulcerative colitis (UC) and Crohn’s disease (CD) [30] (see S1 Table). Sample SRS016585 from the USA group was excluded from the analysis due to an extreme fraction of *Escherichia coli* (possible contamination). Additionally, analysis of the crAssphage presence was performed for 3 new metagenomes from the Russian population obtained using alternative sample preparation protocols (see “Alternative sample preparation protocols”).

To demonstrate the applicability of our method we estimate the relative abundance of genes in five following clinically relevant groups:

1. genes encoding determinants of antibiotic resistance;
2. genes of phage terminases used as marker genes for phage detection in gut microbiota;
3. crAssphage genes;
4. RiPPs biosynthesis gene clusters;
5. genes encoding starch-degrading CAZymes.

Antibiotic resistance gene sequences were taken from the ARDB database [31]. Overall, 7828 genes were divided into 95 resistance type groups according to the ARDB annotation. Phage terminase large subunit genes were downloaded from NCBI (10,177 sequences). Nucleotide sequences of crAssphage genes (*n* = 80) were downloaded from GenBank (ID: JQ995537). The RiPPs biosynthesis cluster gene sequences were extracted from the annotated bacterial genomes described in the original article [7] downloaded from Ensembl (http://www.ensembl.org/) and JGI (http://jgi.doe.gov/). The RiPP cluster abundance analysis was modified to make it similar to the original analysis as follows [7]: non-biosynthetic genes were excluded; a cluster was considered present in a metagenome if the total length of the mapped reads was ≥ 500 bp for at least 50% of its genes; any cluster with coverage higher than 50,000 bp was assigned the value of 50,000 bp; and the relative abundance of a cluster was calculated as an average abundance level of its genes. Concerning the sequences for genes encoding starch-degrading CAZymes, only the complete amino acid sequences of the GH13, GH14 and GH57 families were downloaded from the Carbohydrate-Active enZYmes database (http://www.cazy.org/), resulting in a total of 22,322 sequences. For 13,316 of these sequences, EC numbers were assigned according to the GenBank annotation.

### Alternative sample preparation protocols

The samples from the main healthy group of the Russian cohort were prepared as previously described [29]. Additional samples were collected from healthy Russian individuals (the Ethics Committee of the Research Institute of Physico-Chemical Medicine approved the study protocol, and all participants provided written informed consent). The samples were prepared using dedicated protocols as follows.

- Samples *Lib_15* and *Spb_105*: Frozen fecal samples (150 mg) were combined with guanidine thiocyanate solution (pH 7.4) in a proportion of 100 μl of GTC to 10 mg of sample. Then, the samples were homogenized by vortexing and centrifuged for 5 min at 14,000 rpm. The supernatants were collected in clean tubes and incubated for 5 min at 56C. After cooling to room temperature, a ${}^{1}{/}_{5}$ volume of 96% ethanol was added to each tube and mixed. Samples were transferred to microcolumns (Technoclon, Russia) and centrifuged at 14,000 rpm for 1 min. The filtered solution was discarded. The microcolumns were washed with 300 μl of 80% ethanol and centrifuged twice for 1 min at 14,000 rpm. The ethanol remnants were eliminated by centrifugation. DNA was eluted in 100 μl of deionized water after 5 min of incubation followed by 1 min of centrifugation at 14,000 rpm. The elution was repeated a second time with the filtered solution.
- Sample*Lib_15_Q*: Total DNA was extracted from the frozen fecal samples using the Stool Mini Kit (Qiagen, Germany).

### Figure preparation

Figs 1, 5 and S8, S9 and S11 Figs were plotted using ggplot R package [32]. Figs 2, 4 and 6 and S1, S3, S4, S5, S6, S7 and S10 Figs were plotted using standard R plotting routines [33]. Fig 3 and S2 Fig was plotted using beeswarm R package [34].

## Results and discussion

We developed a pipeline to assess the relative abundance of a user-defined group of genes in human gut metagenomes. Briefly, the approach consists of finding genes similar to the targeted group in a global gut microbial gene catalogue and extracting the abundance values for the detected matching genes basing on a precomputed mapping of metagenomic datasets on the global catalogue (see Methods). We assessed the performance and accuracy of our method as well as determined its applicability and limitations. Also, the developed method was applied to 784 gut metagenomes obtained in several large-scale national studies to explore global variations of several groups of genes of high clinical importance. The gene groups included antibiotic resistance determinants, phage genes [of one selected phage (crAssphage) and all phages), genes involved in biosynthesis clusters, and genes involved in starch metabolism. In addition to the healthy Danish, American, Chinese, Spanish and Russian populations, the analysis was applied to the metagenomes of obese Danish individuals and Spanish patients with inflammatory bowel diseases in an attempt to illuminate the detailed functional features of the microbiota in these important diseases.

### Algorithm validation

#### Test of algorithm accuracy

We evaluated the precision of our algorithm using simulated metagenomic data with known compositions. The relative abundance distributions for 341 reference bacterial genomes (S2 File) were estimated from a published dataset of real gut metagenomes from the Chinese population [28]. The R package HMP [35] was used to generate random read counts from these distributions; then, the read counts were inputted to MetaSim [36] to produce 20 metagenomic readsets.

During the comparison, the relative abundance levels in the simulated metagenomes were calculated for a gene group of ribosomally synthesized and post-translationally modified peptides (RiPPs) biosynthesis gene clusters (*n* = 16,363 gene sequences) using alternative method via direct mapping to a reference frequently used in metagenomic studies [22] [37]. Also, we have compared both methods’ results with an a priori information about the genes abundance in simulated metagenomes (“ground truth”).

To obtain the “ground truth” gene abundances in simulated metagenomes, open reading frames (ORFs) were identified in the reference genomes using PROKKA [38]. Among them, the genes similar to the sequences of the target gene group were detected using BLASTn (percent identity > 95%, alignment length/query length > 0.9, and alignment length/subject length > 0.9). Overall, 8612 genes belonging to 108 gene clusters were found in at least one metagenome. The relative abundance of each gene in a simulated metagenome was defined as the relative abundance of the respective genome.

To calculate gene abundances via direct mapping to the reference, the simulated metagenomic reads were mapped to the sequences of the target gene group using Bowtie (parameters: -t -f -v 3 -k 1). The relative abundance of each gene in a simulated metagenome was defined as the total length of the mapped reads normalized by the gene length.

We calculated correlations between the “ground truth” and each of our catalogue-based method and direct mapping method using the relative abundance data over a range of control parameter values, including the percent identity threshold (see S1 File). First, the catalogue-based method with the set of values used throughout our study was in good correlation with the “ground truth” (Spearman correlation coefficient *r* = 0.68). This result was comparable with the correlation between the direct mapping and the genome-based method (*r* = 0.73). The values of the correlation coefficient between all methods are listed in S3 File.

#### Algorithm performance

To obtain a benchmark for our method, we compared the relative abundance analysis of a sample gene group using our method and a similar procedure performed via direct mapping of reads to gene sequences. The sample gene group included 1000 random amino acid gene sequences from the ARDB database; the test data included 179 MetaHIT readsets (22 mln reads on average, 4.6 Gb in total). The evaluations were performed on a single cluster node (CPUs: 2.30G (24 cores), memory: 62.91G). The wall clock run time statistics were as follows:

1. Direct mapping
  - Construction of a Bowtie index for the gene group sequences: 1 s;
  - Mapping of all reads against the gene group reference: 30,400 s;
  - Processing of all SAM files to yield abundance tables: 148,600 s.
2. Our catalog-based method
  - Construction of a Bowtie index for the global gene catalogue: 9000 s;
  - Mapping of all reads against the global gene catalogue: 109,200 s;
  - Processing of all SAM files to yield abundance tables: 250,600 s;
  - Alignment of the gene group against the global gene catalogue: 1600 s.

Therefore, in a typical study with 10–100 metagenomes, our method becomes computationally more efficient than direct mapping for a gene group of moderate size (n ∼ 1000 genes) due to the exploitation of pre-computed data (see Table 1). Because the alignment of one gene group takes much less time than mapping against it, the analysis time increases insignificantly when more gene groups are compared by direct mapping. Thereby, our method advantages emerge in gene-centric metagenomic studies, where multiple comparisons of targeted sets of genes abundance among metagenomic group are needed. The computational advantages of the proposed method emerge in gene-centric metagenomic studies where the focus in on reduced set of genes rather than global metabolic potential.

**Table 1:** Methods comparison table.

Number of genes in gene group	Total computation time using direct mapping	Total computation time using catalogue-based method
0 (precomputation)	∼ 1 s	∼ 368,800 s
1000	∼ 179,000 s	∼ 370,400 s
2000	∼ 358,000 s	∼ 372,000 s
3000	∼ 537,000 s	∼ 373,600 s
4000	∼ 716,000 s	∼ 375,200 s

Comparison of the wall clock run time between the proposed algorithm (including the pre-computation time) and direct mapping: linear extrapolation based on two values (n = 0 and n = 1000). Linear prediction is suitable because the time required for the sequence alignment is approximately proportional to the size of the query sequence set (and hence the number of gene groups).

#### Representativeness of gene catalog

To evaluate the representativeness of the catalog and its effect on the results, for 138 samples in USA cohort [27] we selected the reads that failed to map to the gene catalog and aligned them directly against the sequences of all the 5 specific gene groups. The alignment was performed: in case of nucleotide sequences—using Bowtie with the parameters used in the manuscript, and in case of amino acid sequences—using DIAMOND with the parameters similar to the ones used with Bowtie (3 mismatches allowed, best hit, random assignment in case of multiple best hits). For each geneset and for each metagenome, we calculated the following discrepancy ratio: (the number of the reads unmapped to the catalog that mapped to the gene set directly) / (the number of the reads that mapped to the catalog). The table below (Table 2) shows the median and standard deviation of the discrepancy ratio across all metagenomes, for each gene set. Discrepancy ratio distribution across gene groups is shown on S2 Fig.

**Table 2:** Distribution of discrepancy ratio for each gene set.

Gene group	Median value of the discrepancy ratio	Standard deviation of the discrepancy ratio
ARDB	0.0047	0.0031
CAZymes	0.0174	0.0130
CrAssphage genes	0.0865	0.0472
Phage terminases	0.0369	0.0440
RiPPs clusters	0.6860	0.6887

For each geneset discrepancy ratio is the number of the reads unmapped to the catalog that mapped to the gene set directly divided by the number of the reads that mapped to the catalog.

As seen, for almost all of the gene groups the value is low, signifying that our catalogue-mediated method captures the majority of the reads related to the genes of interest. The only exception appears to be the case of RiPPs biosynthesis clusters, for the genes of which quite a few of the unmapped reads are still mappable to the gene group directly. We suggest that it is due to the fact that this group of genes was underrepresented in the gut metagenomes of the subjects from the original MetaHIT study. To estimate how this effect impacts the results of the analysis in our manuscript, we compared the RiPPs cluster abundances calculated using 2 methods: 1) via the catalog coverage and 2) via direct coverage of the gene set with the unmapped reads. The correlation value was quite high (Pearson *r* = 0.62) suggesting that the effect does not significantly affect the proportions between the RiPPS clusters.

### Algorithm application

#### Antibiotic resistance genes

**Fig 1:**
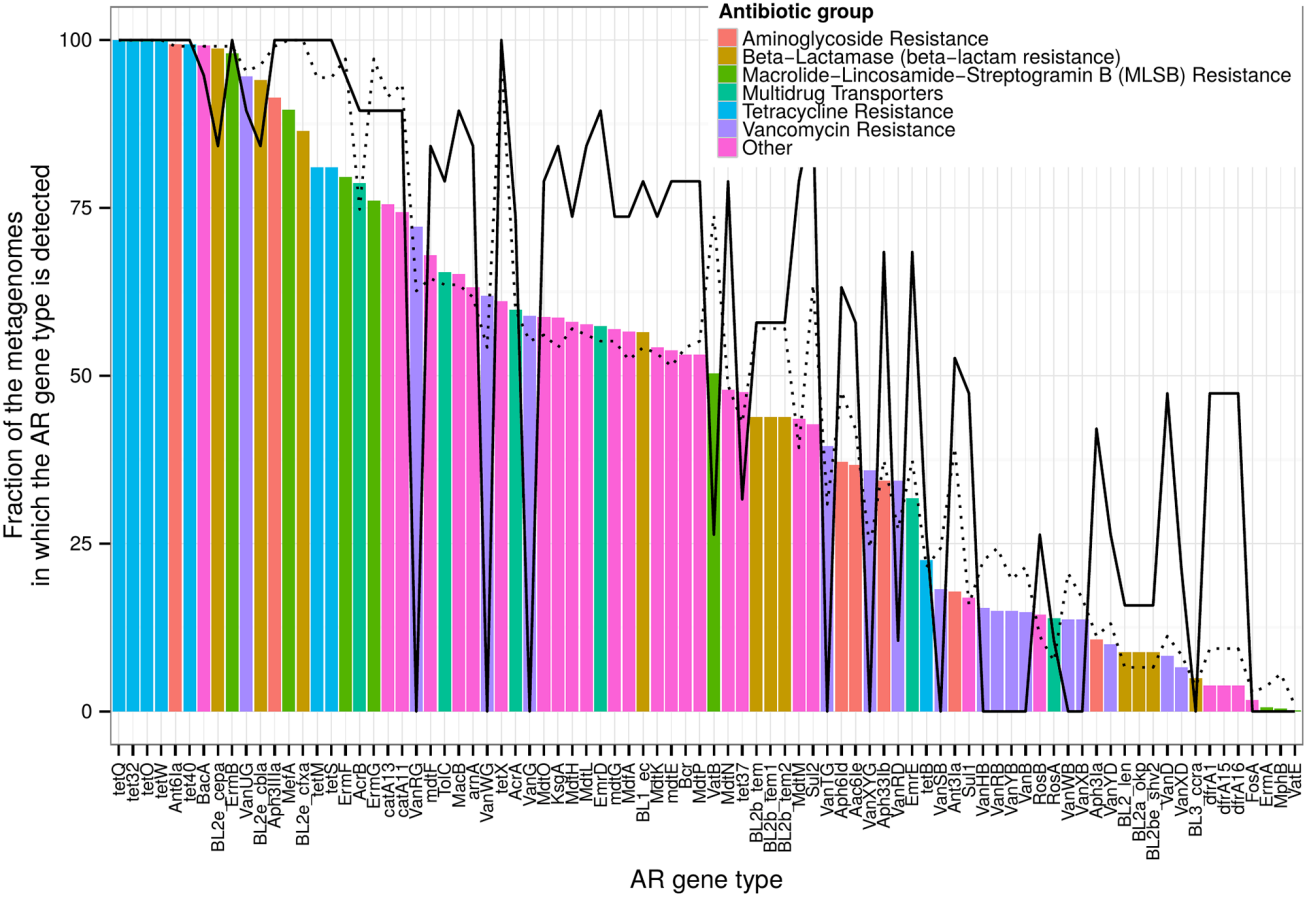
The most prevalent antibiotic resistance gene types in gut metagenomes. Colored bars show the prevalence for the healthy populations of the world. The dotted black line corresponds to fraction in patients with ulcerative colitis only and the solid black line corresponds to fraction in patients with Crohn’s disease only.

Mapping of the sequences from the ARDB [31] database to the global gut microbial gene catalogue led to the identification of 84 similar genes (see Methods). Profiling of these genes in the worldwide metagenomes showed that the most prevalent gene types were related to tetracycline resistance (tet32, tetO, tetQ, tet40 and tetW, see Fig 1). Their omnipresence was in accordance with published results [21] and was likely linked to the fact that tetracycline was one of the first introduced and most widely applied antimicrobial substances. The mentioned gene types conferred bacterial resistance to tetracycline by coding specific proteins that protected the ribosome from the drug. Other frequently occurring genes encoded beta-lactamases (BL2e_cepa, BL2e_cfxa and BL2e_cbla). These genes are inherently present in the genomes of *Bacteroides* spp., which were highly abundant in the examined metagenomes. Among the other prevalent genes were BacA (resistance to bacitracin), Ant6Ia (streptomycin) and ErmB (macrolides, lincosamide, and streptogramin B).

The Russian and Chinese metagenomes had the most diverse resistomes, harboring on average 52 and 48 resistance types in comparison with the 38 each for the Danish and American cohorts. This phenomenon is mirrored at the quantitative level; the group comparison of resistome abundance showed that the Chinese and Russian metagenomes had the highest total levels of AR genes among the healthy individuals (S3 Fig). The respective median relative abundance values were 1.8e-5 and 1.6e-5, which were 2–3 times higher than the values obtained for the Danish and American cohorts (6.6e-6 and 9.9e-6, respectively). Increased levels of gut resistome in Russian and Chinese populations might be caused, in part, by relaxed antibiotic regulations, frequent prescription of wide-spectrum antibiotics as well as over-the-counter availability of the antibiotics [39] [40]. However, a comparison of the relative abundance values between the countries (patients excluded) showed that there was no clear clustering by geography (ANOSIM: *R* = 0.325, *p* = 0.001, see S4 Fig).

Interestingly, the median total level for the Spanish CD metagenomes was also high (1.5e-5) compared not only with the Spanish controls but also with the combined world populations; this effect possibly reflects the consequences of antibiotic therapy (*p* = 0.03 and 0.004, Wilcoxon rank-sum test). The CD metagenomes are particularly distinguished by the profile of resistance to vancomycin; the increased prevalence of the VanD gene was coupled with the decreased abundance of the other Van genes. However, resistance to vancomycin is not determined by a single gene but rather by an operon that is responsible for the synthesis and modification of certain peptidoglycans [41]. In contrast, the Spanish UC metagenomes showed no significant differences in resistome levels compared to either the Spanish control group or the world population (*p* = 0.23 and 0.75, Wilcoxon rank-sum test). The obese Danish individuals were not different from the Danish control group based on their resistome levels (*p* = 0.92).

The AR gene profiles computed using our method are in accordance with previous results obtained for the same Danish, Spanish and Chinese metagenomic datasets by other researchers using their own established microbiome gene sets [22] (pairwise Spearman correlation *r* = 0.66 ± 0.06, mean ± s.d., *p* ≤ 5*e* − 12). To validate the results of our method, we applied an alternative algorithm to assess the relative abundance of the resistome via the direct mapping of metagenomic reads using RAPSearch [42] to the sequences of the ARDB database without the global gene catalogue as a mediator. The results of both methods were highly correlated (paired Pearson correlation of AR gene types levels *r* = 0.923 ± 0.003, *p* = 0 for all). Therefore, this example supported the applicability of our method as an efficient routine for profiling a user-defined gene group in the gut metagenome by exploiting reads pre-mapped to the global gene catalogue without the need for mapping to each new group of genes of interest (ARDB database here).

#### CrAssphage

Our method is not limited to bacterial genes that normally form the majority of the total gut metagenome but can also be applied to the genes of other organisms, including bacterial viruses (phages). Recently, a dominating gut viral component (crAssphage) was identified in gut metagenomes from multiple studies [37]. To evaluate our method for viral gene profiling using this major representative and to expand the understanding of the worldwide spread of this phage, we applied our pipeline to estimate the relative abundance of its genes (*n* = 80) in the gut metagenomes of the worldwide population (see Methods). Out of 80 crAssphage genes, 79 were found in the reference catalog with average identity of 92%.

**Fig 2:**
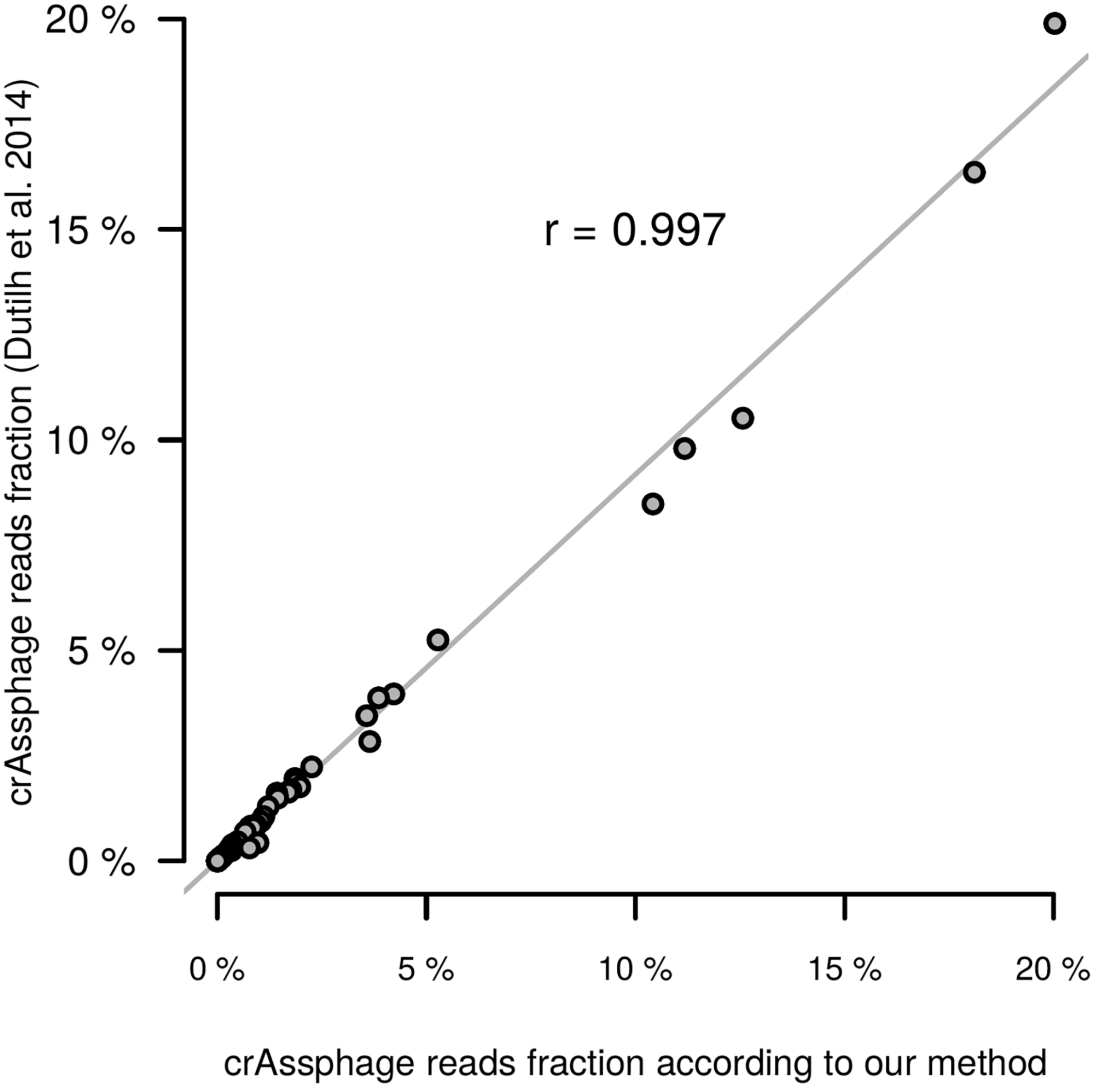
Comparison of the crAssphage abundance obtained for the USA group using our method with the previously published results. Each dot represents one sample. The X-axis represents the crAssphage reads fraction in this sample according to our method, the Y-axis—according to data from Dutilh et al, 2014.

For the validation of our approach, we examined the American metagenomes because the crAssphage presence in these metagenomes was also assessed by the authors of the original article using an alternative method (mapping of the reads to the phage genome) [37]. Our results were highly correlated with the published results (for 71 metagenomes with at least one crAssphage gene detected: Pearson correlation *r* = 0.997, *p* = 0, see Fig 2).

**Fig 3:**
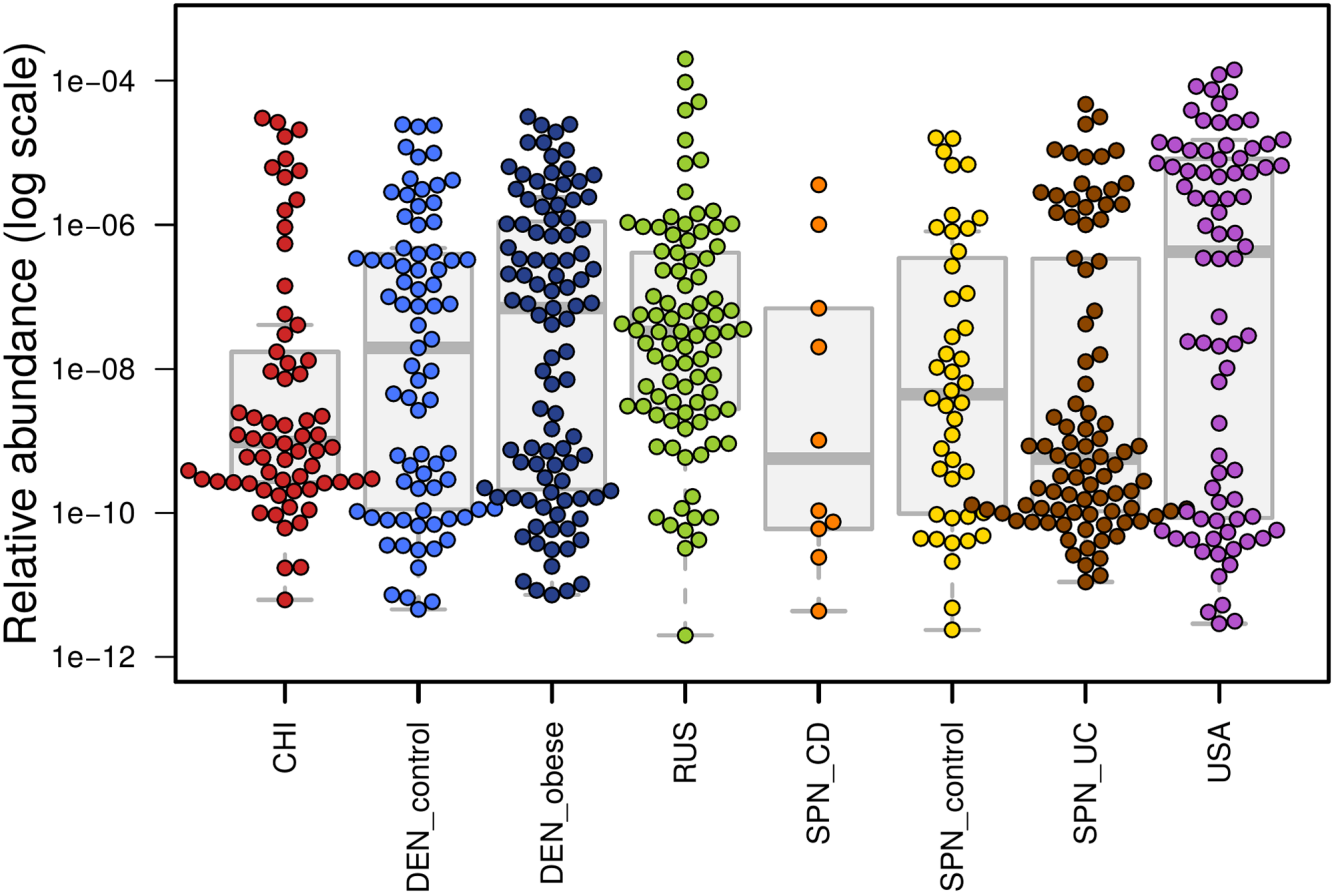
Relative abundance of crAssphage reads in groups. Colored points represent distinct samples in different groups, with group statistics represented by faded boxplots in the background. Samples where no crAssphage genes were detected are not shown.

Further analysis showed the presence of the crAssphage in the metagenomes of other populations, including Russians; however, the levels were lower than the levels observed in the American metagenomes (4.7e-7–2.2e-6 vs. 1.1e-5 on average across the healthy controls, Fig 3), indicating the global phylogeography of the phage.

In addition to the main set of Russian metagenomes, the analysis of the crAssphage presence was also performed for a set of previously unpublished metagenomes of the Russian population obtained using various sample preparation protocols. Several of these analyses produced interesting observations. First, a sample with an unusually high level of crAssphage was identified (ID: Lib_15, 24.51% of total reads, Table 3). To evaluate the influence of the sample preparation protocol, we compared the result with the same sample prepared using an alternative experimental procedure (ID: Lib_15_Q, see Methods). Interestingly, the fraction was decreased almost two-fold, possibly indicating significant variation of the extractability of the viral fraction across the protocols (although more samples are required for statistically grounded conclusions). One of the known effects of the choice of DNA extraction method on the obtained taxonomic composition of a metagenome is linked to varying efficiency of cell wall lysis. Particularly, it can lead to varying ratio of gram-positive (mainly represented by *Bacteroidetes* and *Actinobacteria* phyla in the human gut) to gram-negative bacteria (mainly *Firmicutes*). The exact bacterial host(s) of crAssphage has not been identified so far, but it is assumed to belong to *Bacteroides* genus (within *Bacteroidetes* phylum). Taxonomic analysis using Metaphlan2 [43] showed that the levels of *Bacteroides* were somewhat decreased with Stool Mini Kit method: Lib_15 contained 73% of *Bacteroides* (with crAssphage read fraction 24.5%) and Lib_15_Q—59% (crAssphage: 12%). Supposedly, a higher level of the phage in Lib_15 metagenome could be linked to a possibly better cell wall lysis for *Bacteroides* under the respective protocol (thus the phages contained within the cell would be extracted more efficiently)—but not explaining the 2× change in the phage levels. The Qiagene Stool Mini Kit is a closed format so a more detailed comparison of the reagents used in that kit and our second method—and of the consequent variations in extraction efficiency—is not possible. Further analysis on a higher number of samples prepared using 2 methods would yield more statistically sound conclusions on the difference between the methods.

**Table 3:** Metagenomes containing high levels of the crAssphage reads.

Metagenome ID	Fraction of the crAssphage reads according to our method, % of total reads	Cohort
Lib_15	24.51	RUS
Lib_15_Q	11.97	RUS
O2.UC36-2	6.03	SPN_UC
Spb_105	5.98	RUS
NOV_282	4.89	RUS
SRR413594	3.83	CHI
O2.UC20-0	3.74	SPN_UC
MH0128	3.68	DEN_obese
SRR413585	3.37	CHI
MH0462	3.13	DEN_obese

Our results for crAssphage abundance in non-USA metagenomes. See S4 File for all samples.

Second, the list of the Russian metagenomes enriched in crAssphage included 2 time points from the same healthy individual (0.5 year lapse). Although these samples were also prepared using different protocols, the observation is evidence of a temporal persistence of crAssphage in the human gut microbiota.

Analysis of the patients’ metagenomes showed that crAssphage did not have any strong association with clinical status. The crAssphage levels did not differ between the Spanish controls and the CD group (*p* = 0.06) and UC group (*p* = 0.64); this finding was also observed for the Danish controls and obese subjects (*p* = 0.88). These results suggest that the phage is not likely to be directly involved in the pathogenesis of these disorders.

#### Bacteriophages

Overall, the role of phages in the human gut is currently the focus of active research. Phages are highly diverse and can carry non-viral “cargo genes” to increase the fitness of their bacterial hosts and thus affect the ecology of the whole community [44]. Evidence of the association of phages with host disease has started to appear. For example, patients with inflammatory bowel diseases demonstrate an increased abundance of phages coupled with a decrease in microbial diversity [45]. Here, we applied our method to assess the presence of phages using genes encoding terminase large subunits, which are involved in viral DNA packaging, as viral phylogenetic markers in the gut metagenomes (see Methods). This gene was previously used as a phage marker [46]. However, the use of this gene has some limitations: terminases are not detected in all phages and are best described for the *Caudovirales* order (tailed phages). Additionally, taxonomic annotation of terminases often refers to the phage host taxonomy rather than to the phage taxonomy. Obviously, our method does not allow us to distinguish between the presence of viral particles and the prophage form.

**Fig 4:**
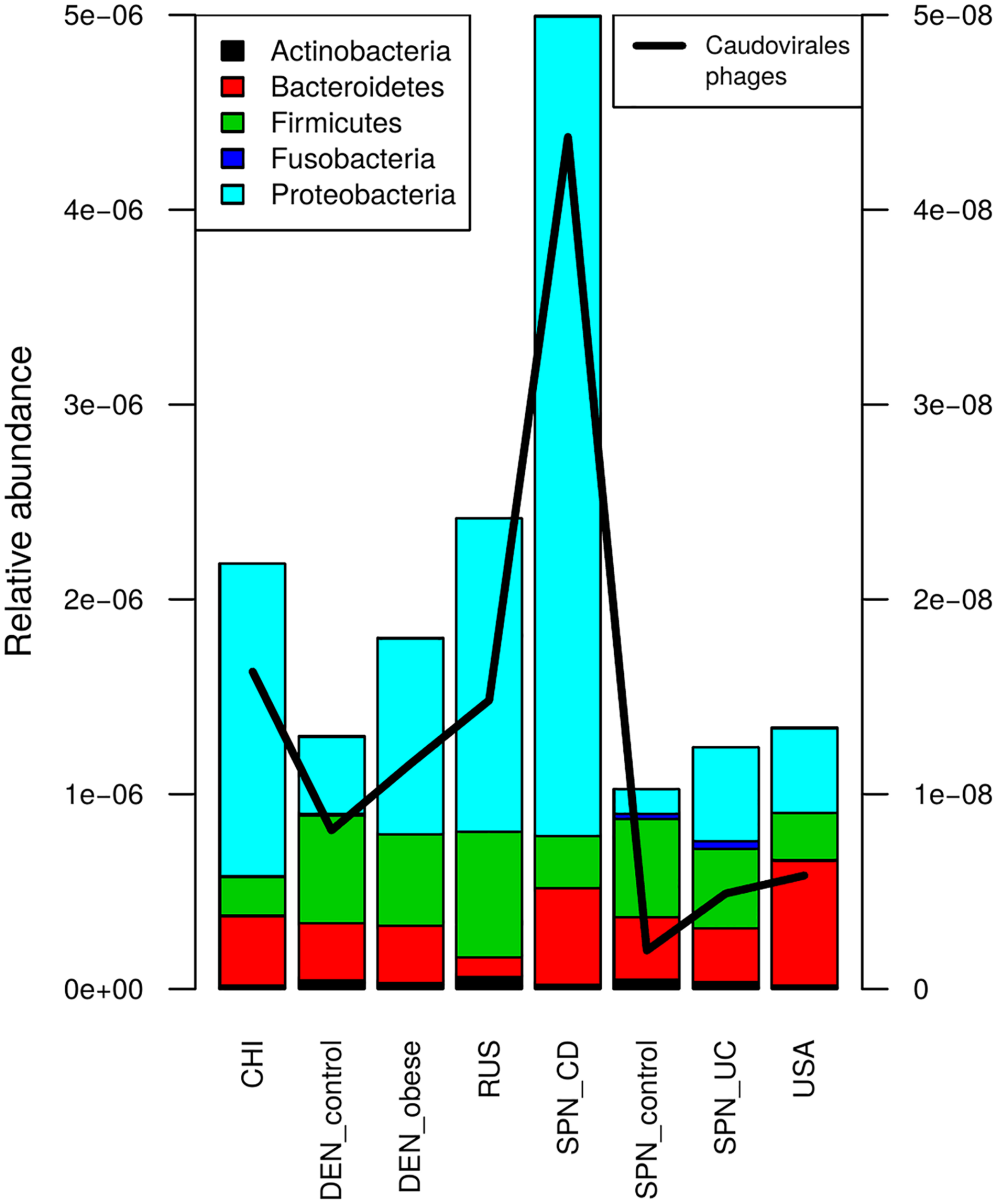
Taxonomic diversity and mean abundance of phages. Stacked bars indicate denote abundance of phages with different host taxonomy (represented with color) in the gut metagenomes according to the terminase large subunit profiling (left vertical axis). The relative abundance of tailed phages is shown separately as a black line (right vertical axis).

The total relative abundance values for phages in the worldwide cohorts are summarized in S5, S6 and S7 Figs. There were no significant differences in the total phage gene levels between the healthy populations. Interestingly, the Spanish CD group carried a higher fraction of phage reads compared to the healthy Spanish group and the other healthy groups (*p* < 0.05, Wilcoxon rank-sum test). This difference appears to be primarily caused by phages of Proteobacteria (Fig 4) because the abundance of this phylum is much higher in the CD samples than in each of the other groups. On a more detailed level, when the analysis was limited to only tailed phages, their levels were also higher in the CD group than in all other healthy groups except for the Chinese (*p* < 0.05, Wilcoxon rank-sum test); this observation was in accordance with recently published findings [45]. Interestingly, the UC metagenomes showed no significant differences in phage levels (S6 Fig). We also did not find a relationship between the phage abundance and BMI (S7 Fig).

#### Biosynthesis clusters: RiPPs

Another gene group of biomedical interest selected for analysis using our method was biosynthesis genes, with the genes encoding RiPPs (ribosomally synthesized and post-translationally modified peptides) used as an example. Recently, a rich diversity of biosynthesis genes in the human metagenome was identified [7]. RiPPs include lantipeptides (antimicrobial agents endowed with great pharmaceutical potential), which are viewed as a replacement for traditional antibiotics.

We assessed the presence of the biosynthesis gene clusters described by Donia et al. (a total of 16,363 genes forming 285 clusters) in the gut metagenomes (see Methods). Overall, 23 of the detected clusters were identified in at least one metagenome.

**Fig 5:**
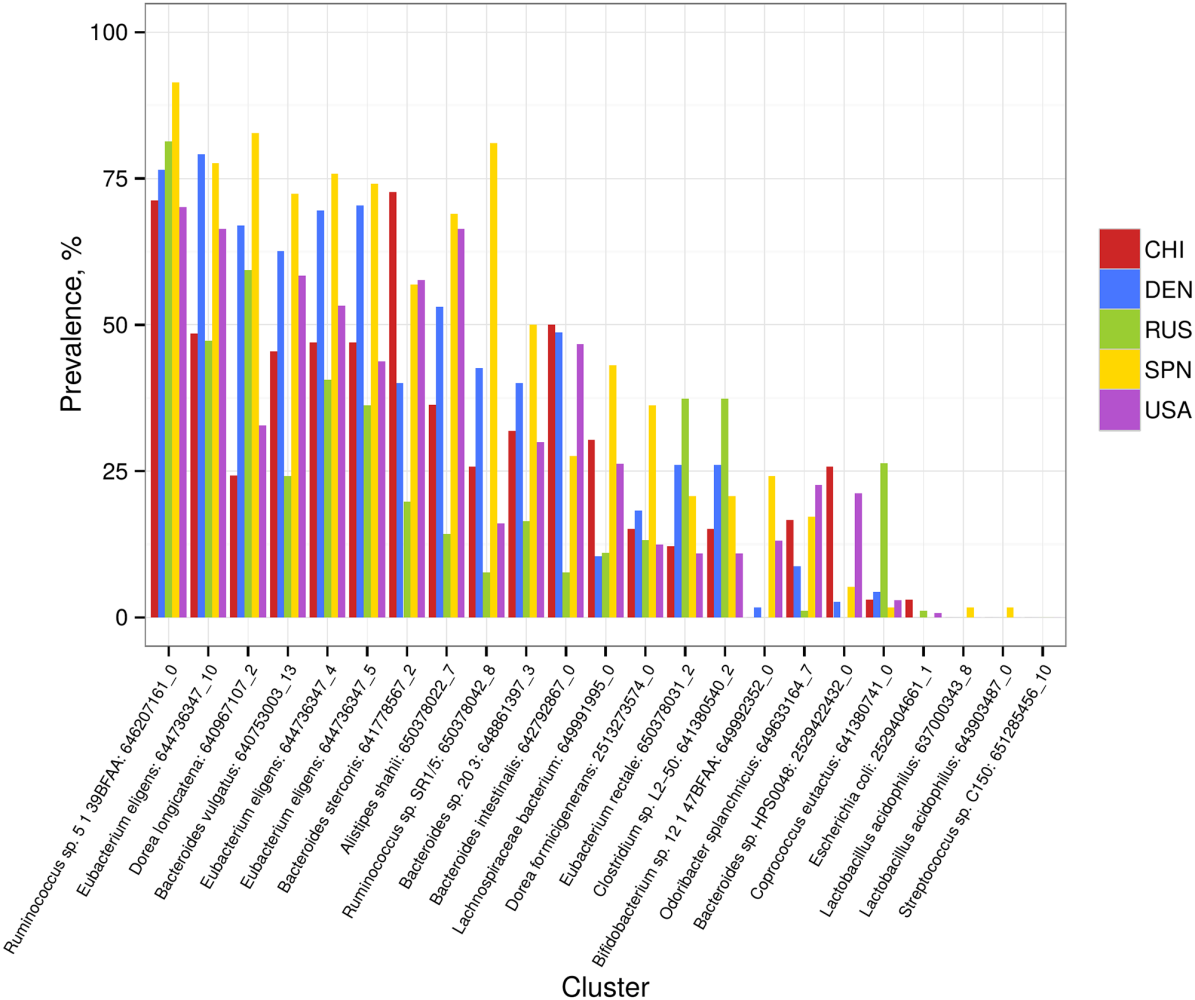
Prevalence of the detected RiPP synthesis clusters in the metagenomes of healthy populations. The cluster names (along X axis) are indicated as in the original article [7]. For each cluster, there are 5 colored bars indicating the prevalence of the cluster for each of the groups.

We observed significant diversity in the RiPP profiles across the healthy populations (Fig 5). No cluster-wise functional annotation was available; however, it was possible to assess the general functional potential for a given cluster from the annotation of its genes. For instance, cluster 641380741_0 belonging to *Coprococcus eutactus* is strongly enriched in Russian metagenomes compared with the other cohorts and includes genes encoding hydrolases, peptidases, ABC transporters, a fibronectin domain, a transcriptional regulator and hypothetical proteins.

Analysis of the RiPP coding capabilities in patients showed that the metagenomes of CD patients were distinguished from the Spanish controls by a pronounced increase in the abundance of two clusters [2513273574_0 (*p* < 0.05) and 640753003_13 (*p* < 0.0005)] (see S8 Fig). Both clusters primarily include genes with unknown functions and genes related to mobile elements. The second cluster belongs to the *Bacteroides vulgatus* species and contains genes involved in carbohydrate metabolism and DNA processing. Comparison of Spanish UC patients with the Spanish controls did not reveal significant differences in the abundance of any of the RiPP biosynthesis clusters; the same result was obtained when obese and non-obese individuals from Denmark were compared (S9 Fig).

#### Starch degradation enzymes

One of the central metabolic activities of gut microbes is the degradation of dietary complex polysaccharides (starch, fiber and others). The final products include short-chain fatty acids, particularly butyrate, which plays an important role in host homeostasis (i.e., as the sole source of energy for epithelial cells and as an anti-inflammatory and anticancer agent). From this perspective, a quantitative metagenomic analysis of the genes involved in these fermentative processes can provide interesting insights. Specifically, we applied our method to assess the genes associated with the degradation of starch, which is a major carbohydrate in the human diet. Various forms of starch remain undigested and reach the colon to be metabolized by gut microbes. These genes encode many catalytic enzymes, including the alpha-amylases that hydrolyze 1,4-alpha-bonds and the pullulanases that hydrolyze 1,6-bonds.

**Fig 6:**
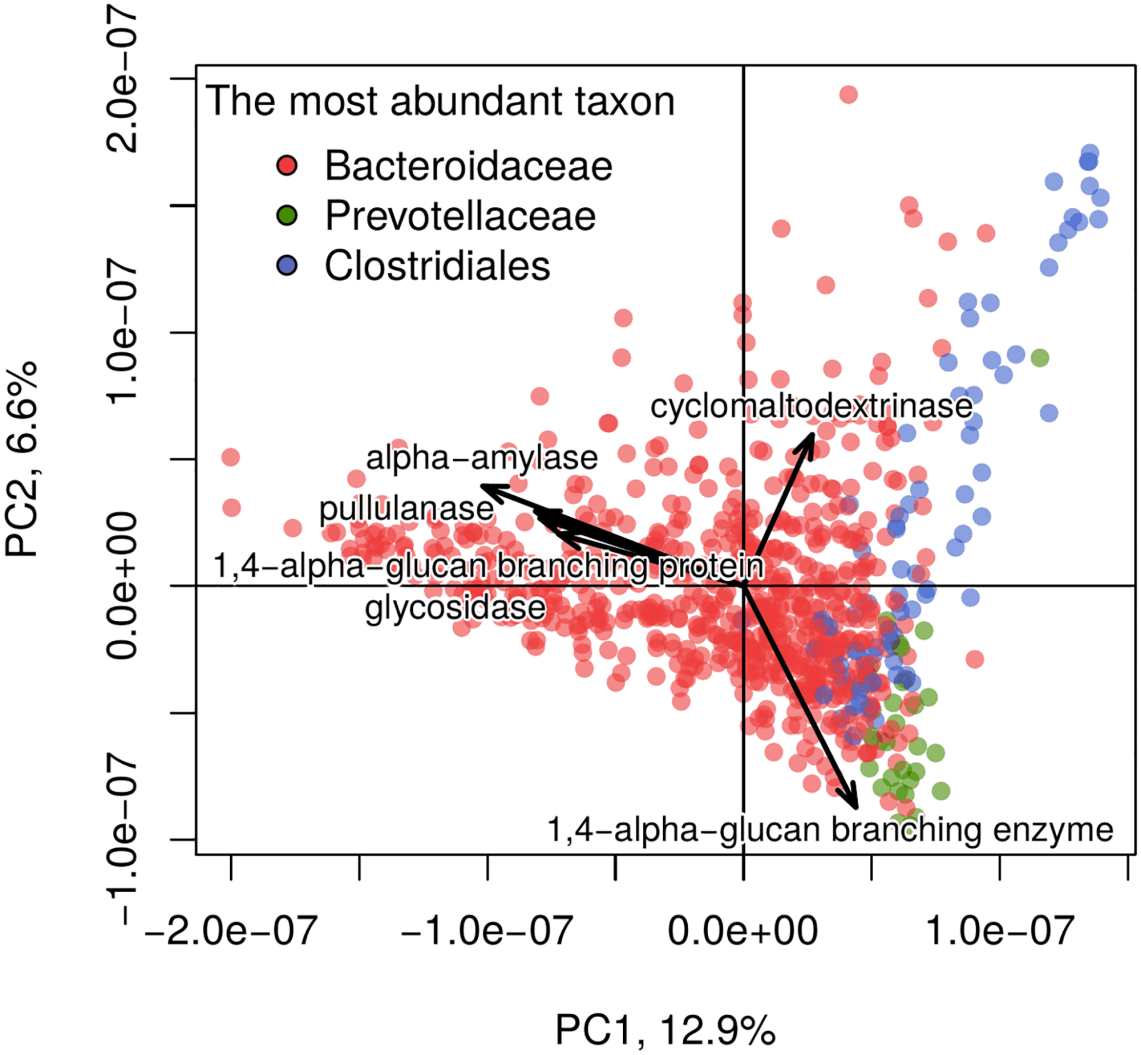
Distribution of the metagenomes by the abundance of the genes associated with starch degradation. On the PCA plot, for each sample, the color shows the most abundant taxon. The color legend represents the three major taxa (the *Bacteroidaceae* and *Prevotellaceae* families and the *Clostridiales* order). The latter taxon was taken at a higher level of phylogenetic hierarchy because it includes multiple starch-degrading families (i.e., *Eubacteriaceae* and *Ruminococcaceae*), which together contribute to the observed gradient without a clear single driver. The percent of total variance explained by each of the first two principal components is included in the respective axes labels.

For the analysis, the GH13, GH14 and GH57 groups containing genes of the majority of the enzymes involved in starch metabolism were obtained from the Carbohydrate Active enZYmes database [47] (22,322 genes in total) and their abundances were determined (see Methods). Overall, 3065 respective genes (corresponding to 529 genes from the reference catalogue) were present in at least one metagenome. No clear clustering by geography was identified, but samples from some groups (e.g. USA and RUS) tended to be shifted to one of the three directions on the PCA plot (S10 Fig). Analysis of the distribution using a biplot revealed distinct genes increasing in the respective directions. The directions were explained by the trade-off between the taxa *Bacteroidaceae* (mainly the *Bacteroides* genus), *Prevotellaceae* (*Prevotella*) and *Clostridiales*, which are the drivers of gut microbiota possessing of prominent fermenting activity [48] (Fig 6).

Although the directions were driven by different genes belonging to microbes from these taxa, their functional categories according to the Enzyme Commission (EC) were similar (see Table 4). Therefore, these genes contributed to the comparable metabolic potential in seemingly distinct metagenomes. Overall, the most dominant gene categories included alpha-amylases (EC 3.2.1.1), pullulanases (EC 3.2.1.41) and 1,4-alpha-glucan branching enzymes (2.4.1.18; see S11 Fig). Although the information on diet followed by the subjects from whom the stool samples were collected is not available, the observed variability in the abundance proportions of the genes reflects the specific metabolic capabilities of individual microbiotas for carbohydrate degradation and could be linked to varying polysaccharide contents in the host diet [49] [50].

**Table 4:** Prevalent genes encoding carbohydrate-active enzymes.

GenBank ID	EC number	EC description	Organism (GenBank)
ABR39071.1	<NA>	alpha-amylase	*Bacteroides vulgatus* ATCC 8482
CBK65614.1	<NA>	glycosidase	*Bacteroides xylanisolvens* XB1A
ABR40948.1	2.4.1.18	1,4-alpha-glucan branching protein	*Bacteroides vulgatus* ATCC 8482
ACR74776.1	<NA>	cyclomaltodextrinase	*Eubacterium rectale* ATCC 33656
ABR40671.1	3.2.1.1	pullulanase	*Bacteroides vulgatus* ATCC 8482
AGB28031.1	3.2.1.1	1,4-alpha-glucan branching enzyme	*Prevotella dentalis* DSM 3688

## Conclusions

Early metagenomic studies were primarily focused on the taxonomic compositions of the microbial communities. However, the same species can carry various gene contents that significantly modulate the ecological role of an organism; in the human gut microbiota, these roles range between the extremes of probiotic and pathogen. These phenomena highlight the importance of functional gene-centric analyses of the metagenome. Selecting individual genes from millions for dedicated quantitative profiling has proven to be difficult. Moreover, the analysis of numerous groups of genes might be too general and not lead to biological conclusions. Ultimately, an optimal level of detailing is often provided by focusing on gene groups involved in specific functions. A comparative analysis of clinically important gene groups in the metagenomes of healthy subjects and patients with various diseases provides a multi-faceted portrait of the dysbiosis and ultimately approaches the understanding of the pathogenesis mechanism.

Here, we presented a method for the quantitative profiling of gene groups in the gut microbiota. Due to the exploitation of pre-computed mapping against a representative microbial catalogue, the method is particularly useful in situations in a metagenomic study of the human microbiota where it becomes necessary to include a few additional genes into the analysis. Our approach allows the avoidance of repeated mapping, thereby decreasing the total overhead. The results were validated using simulated metagenomic data and by comparison with real metagenomic data analyzed by other authors using alternative techniques.

In the example of 5 gene groups with either predicted or proven clinical relevance, we analyzed their abundances in the gut metagenomes of the healthy worldwide populations and patients with diseases that posed global public health challenges. We discovered country-specific functions and distinct features of the microbiota in the diseases. The microbiota in Crohn’s disease demonstrated the highest deviations from the nation-matched healthy population, including a significant increase in antibiotic resistance genes (possibly reflecting the consequences of antimicrobial therapy), high levels of tailed phages and biosynthesis gene clusters with presence of genes with unknown functions. Interestingly, a similar analysis of gut metagenomes in ulcerative colitis showed none of these differences. This discrepancy is likely linked to the fact that although UC is also an inflammatory bowel disease; unlike CD, it commonly affects certain parts of the intestinal tract, is associated with a less extreme gut microbial phenotype [51] and is subject to less frequent antibiotic treatment [52].

Profiling of the genes belonging to a recently discovered major gut bacteriophage (crAssphage) showed that although this phage was present globally, its abundance varied across populations worldwide, with the highest levels in the USA and China. We demonstrated the temporal resilience of crAssphage and the influence of the sample preparation protocol on its levels by processing several new metagenomes from the Russian population. The observed lack of association with the clinical status across nation-matched cohorts suggests that the phage is not involved in the pathogenesis of either IBD or obesity. The observed geographic variation in starch degradation gene levels suggests links to the specifics of national dietary habits.

The database annotation is likely to affect the results of our analysis, but the investigation of this factor is out of scope of our study and is described elsewhere (e.g., [53]). If the sequences of the query gene group were obtained from an incomplete or elsewise deficient database, the estimation of the group abundance will be biased. Also, abundance estimation depends on representativeness of catalog. In this study, analyses of all gene groups were performed based on the gut gene catalogue from the original MetaHIT study [2]. Updated versions of the catalogue have since been published [54] [30], and alternative catalogues have been constructed *de novo* in other studies. The algorithm can be easily adjusted to any of the versions, and generally our observations are not expected to radically change because the expansion of the gene catalogue primarily occurs due to the addition of rare genes. Overall, the developed approach is computationally flexible with regard to the addition of new metagenomes and gene sets and can serve as a convenient tool in gut metagenomic studies.

## Availability of supporting data

The precomputed gene abundance tables and code are available at https://osf.io/m758w. Also, pipeline is available at https://github.com/KonstantinYarygin/Yarygin_2016. The new metagenomic readsets (IDs: *Lib_15*, *Lib_15_Q* and *Spb_105*) are available from the European Nucleotide Archive (ENA) database (http://www.ebi.ac.uk/ena) under accession number ERP015053.

## Supporting information

S1 File Comparison between catalogue-based method, direct mapping method and “ground truth”. Spearman and Pearson correlations of abundance vectors and precision-recall values are calculated for: 1) Direct mapping method vs. “ground truth” 2) Catalogue-based method vs. “ground truth” using various thresholds.
(XLSX)

S2 File List of bacterial species which genomes was used in metagenomes simulation. (TXT)

S3 File Spearman and Pearson correlations of abundance vectors between three methods: Catalogue-based method, direct mapping method and “ground truth”. (XLSX)

S4 File Fraction of crAssphage reads among all metagenomes, used in this study. (CSV)

S1 Table Metagenome groups used in this study. (CSV)

S1 Fig Sensitivity of the proposed method to detect genes with various scale of abundance. Bars show the number of genes with certain abundance (x-axis). Red part of the bars represents fraction of genes detected by the algorithm. For each gene, its abundance was calculated as median read count across 20 simulated samples.
(TIFF)

S2 Fig Discrepancy ratio distribution for 5 gene groups. Each dot represents a value of discrepancy ratio for a gene group in a single metagenome. Samples with zero or infinite discrepancy ratios are not shown.
(TIFF)

S3 Fig Total relative abundance of antibiotic resistance genes in gut metagenomes. The cohorts are sorted in the increasing order of medians.
(TIFF)

S4 Fig Distribution of AR genes profile. Samples are shown as points on multidimensional scaling plot with dissimilarity measure = 1—Spearman correlation. Colors denote sample cohort, lines show convex hull of each cohort.
(TIFF)

S5 Fig Total relative abundance of phage terminases in healthy gut metagenomes. The abundance distribution in groups is shown with boxplots in logarithmic scale. A) Distribution of all phage terminases; B) Distribution of Caudovirales phages terminases only.
(TIFF)

S6 Fig Total relative abundance of phage terminases in gut metagenomes of Spanish individuals. The abundance distribution in groups is shown with boxplots in logarithmic scale. A) Distribution of all phage terminases; B) Distribution of Caudovirales phages terminases only.
(TIFF)

S7 Fig Total relative abundance of phage terminases in gut metagenomes of Danish individuals. The abundance distribution in groups is shown with boxplots in logarithmic scale. A) Distribution of all phage terminases; B) Distribution of Caudovirales phages terminases only.
(TIFF)

S8 Fig Group-wise average abundance of RiPPs biosynthesis clusters in healthy individuals, patients with Crohn’s disease and with ulcerative colitis from Spain. Cluster names along the X axis are indicated as in the original article [7]. For each cluster there are 3 colored bars for 3 sample groups, indicating mean abundance of RiPPs biosynthesis cluster in group.
(TIFF)

S9 Fig Group-wise average abundance of RiPPs biosynthesis clusters in healthy and obese individuals from Denmark. Cluster names along the X axis are indicated as in the original article [7]. For each cluster there are 2 colored bars for 2 sample groups, indicating mean abundance of RiPPs biosynthesis cluster in group.
(TIFF)

S10 Fig Distribution of gut metagenomes by the relative abundance of starch degradation-associated genes (PCA biplot). Arrows show the directions in which the levels of the 6 most abundant genes increase. Lines show convex hull for each cohort.
(TIFF)

S11 Fig Group-wise median abundance of starch degradation genes (EC numbers). For each EC number, there are 5 bars for 5 sample groups indicating mean sum abundance of all genes with corresponding EC number in group.
(TIFF)
