# Supplementary figures and images for: Abundance profiling of specific gene groups using precomputed gut metagenomes yields novel biological hypotheses
Source: PLoS One. 2017 Apr 27;12(4):e0176154. doi: 10.1371/journal.pone.0176154 (PMC5407692; doi:10.1371/journal.pone.0176154)

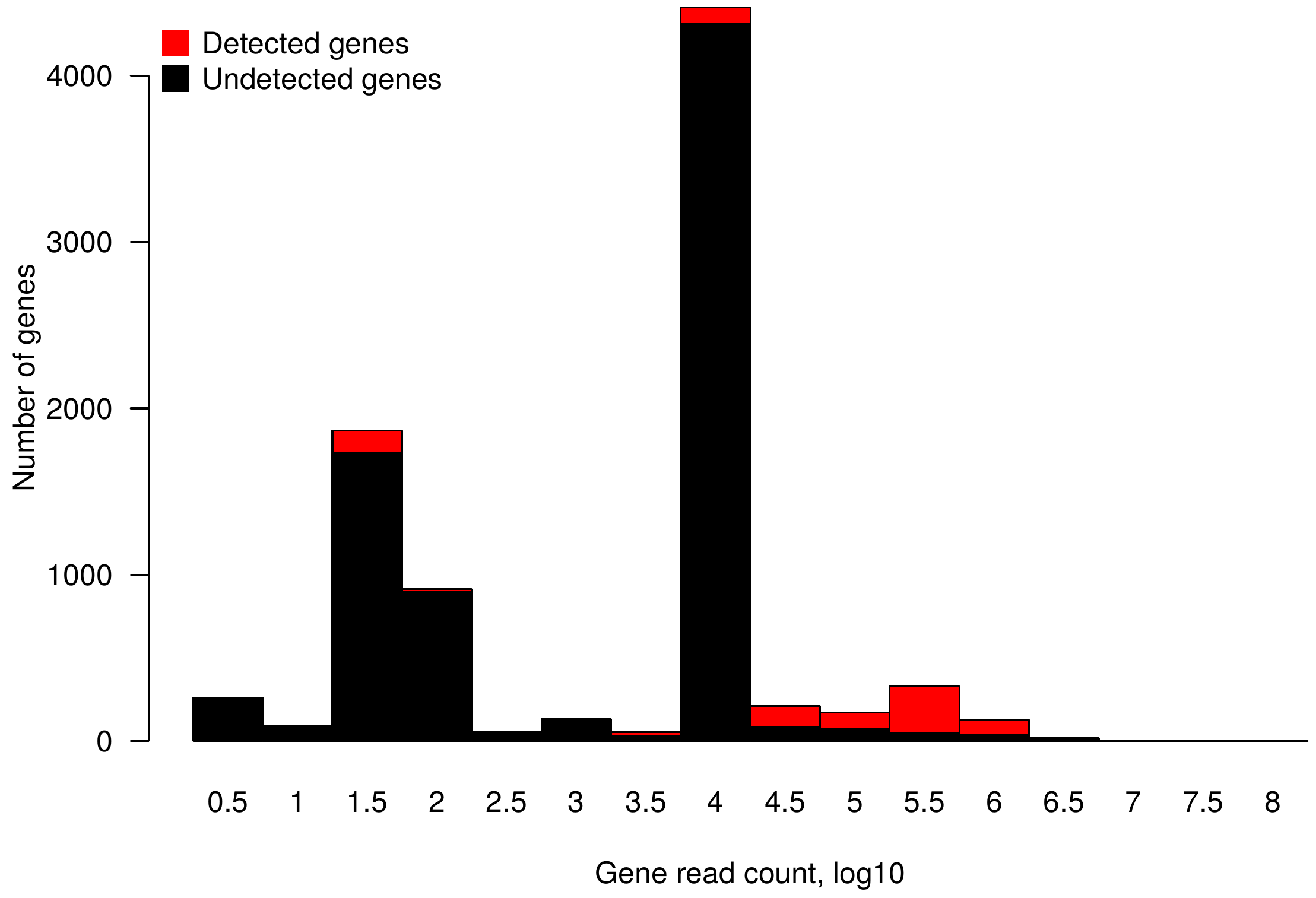

Supplement: S1 Fig — Bars show the number of genes with certain abundance (x-axis). Red part of the bars represents fraction of genes detected by the algorithm. For each gene, its abundance was calculated as median read count across 20 simulated samples. (TIFF) [file pone.0176154.s006.tiff]

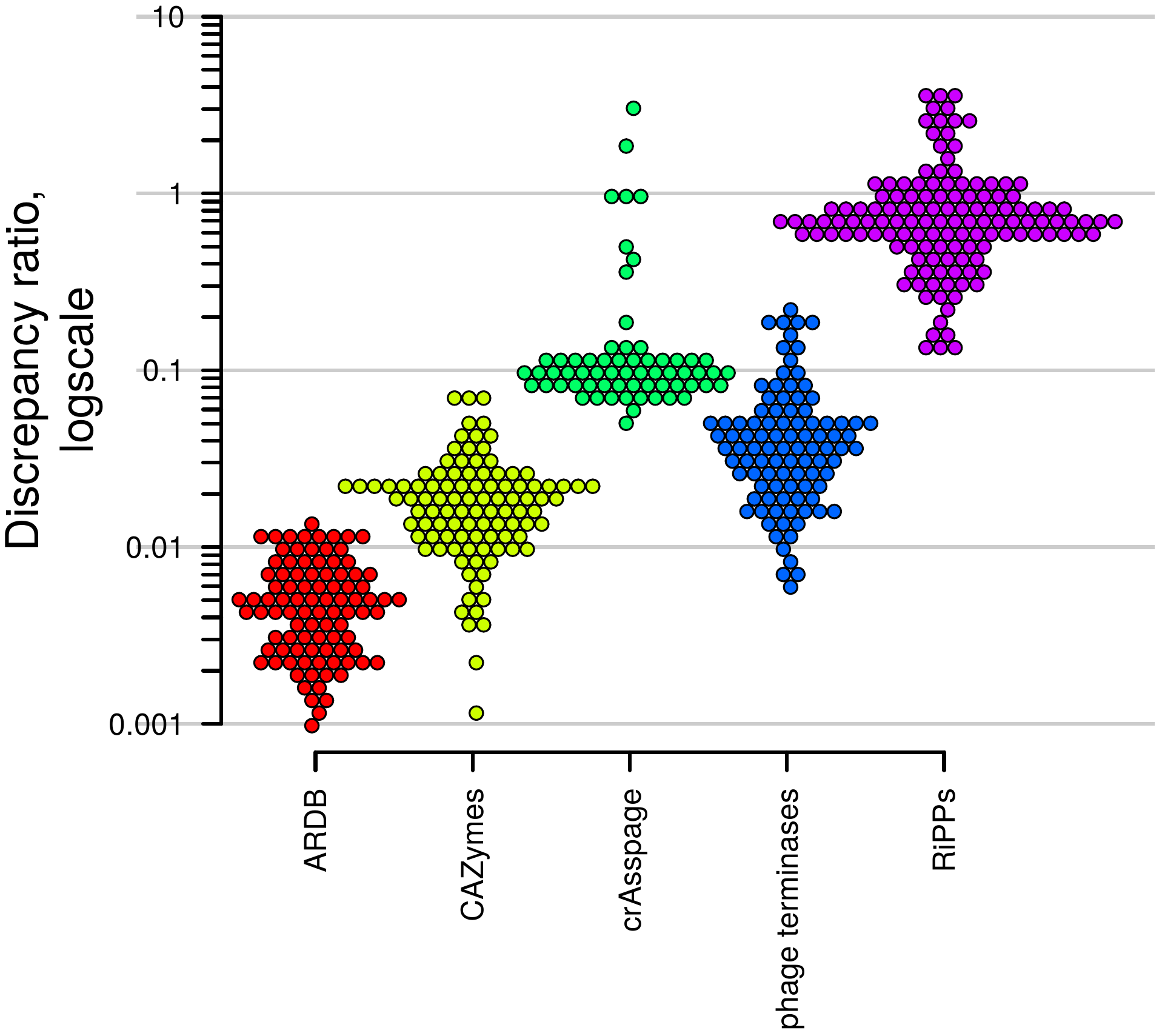

Supplement: S2 Fig — Each dot represents a value of discrepancy ratio for a gene group in a single metagenome. Samples with zero or infinite discrepancy ratios are not shown. (TIFF) [file pone.0176154.s007.tiff]

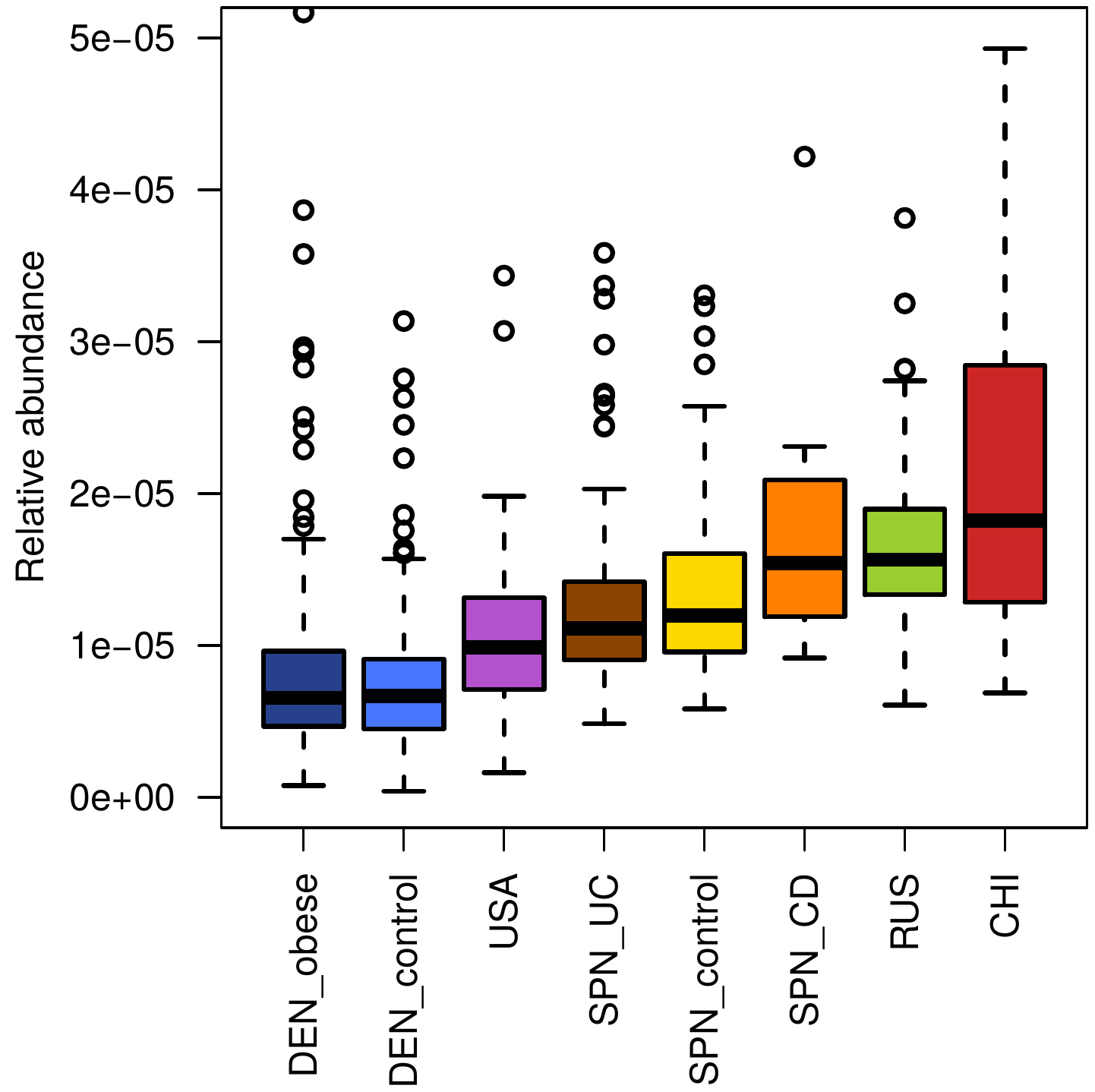

Supplement: S3 Fig — The cohorts are sorted in the increasing order of medians. (TIFF) [file pone.0176154.s008.tiff]

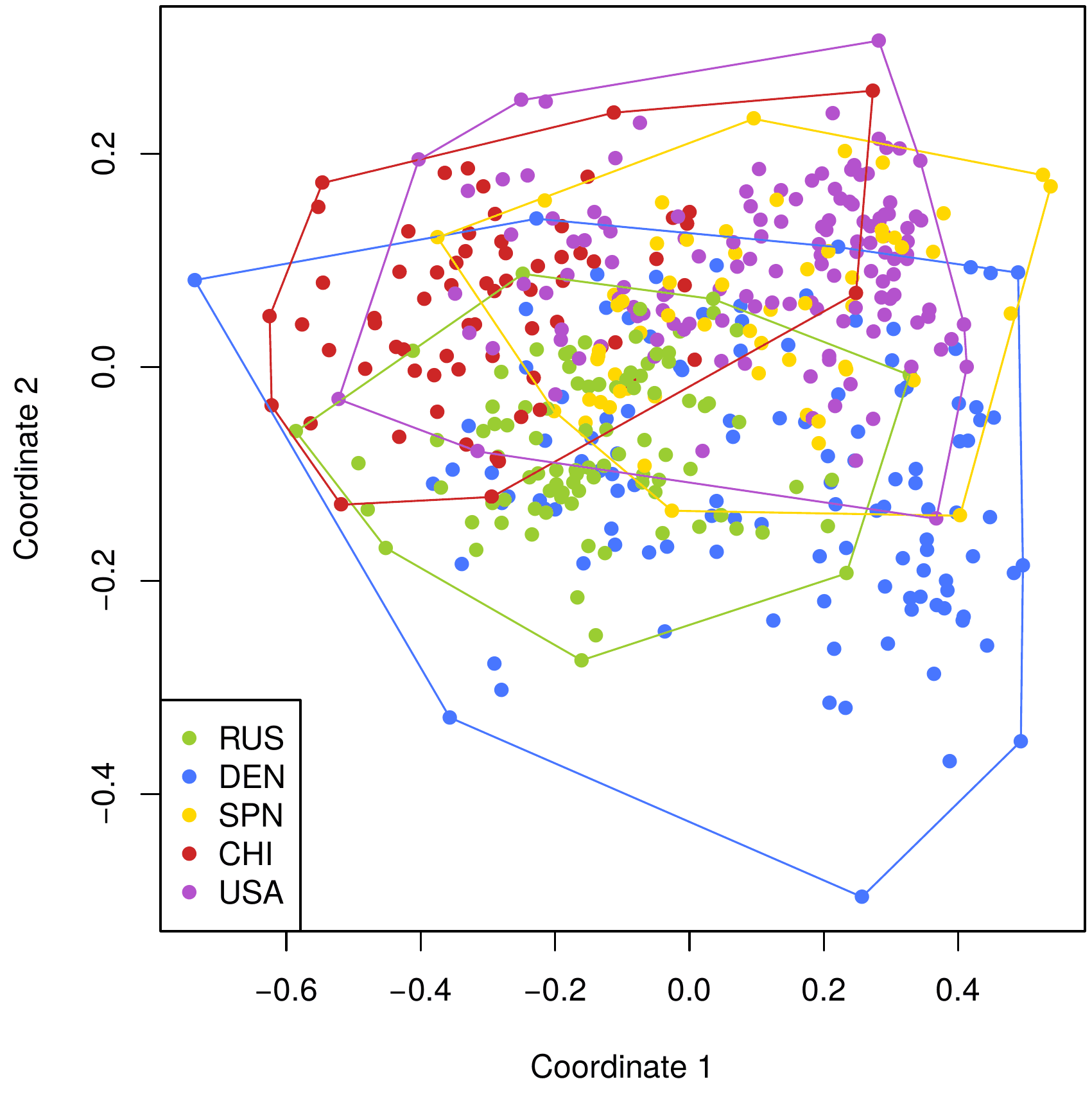

Supplement: S4 Fig — Samples are shown as points on multidimensional scaling plot with dissimilarity measure = 1—Spearman correlation. Colors denote sample cohort, lines show convex hull of each cohort. (TIFF) [file pone.0176154.s009.tiff]

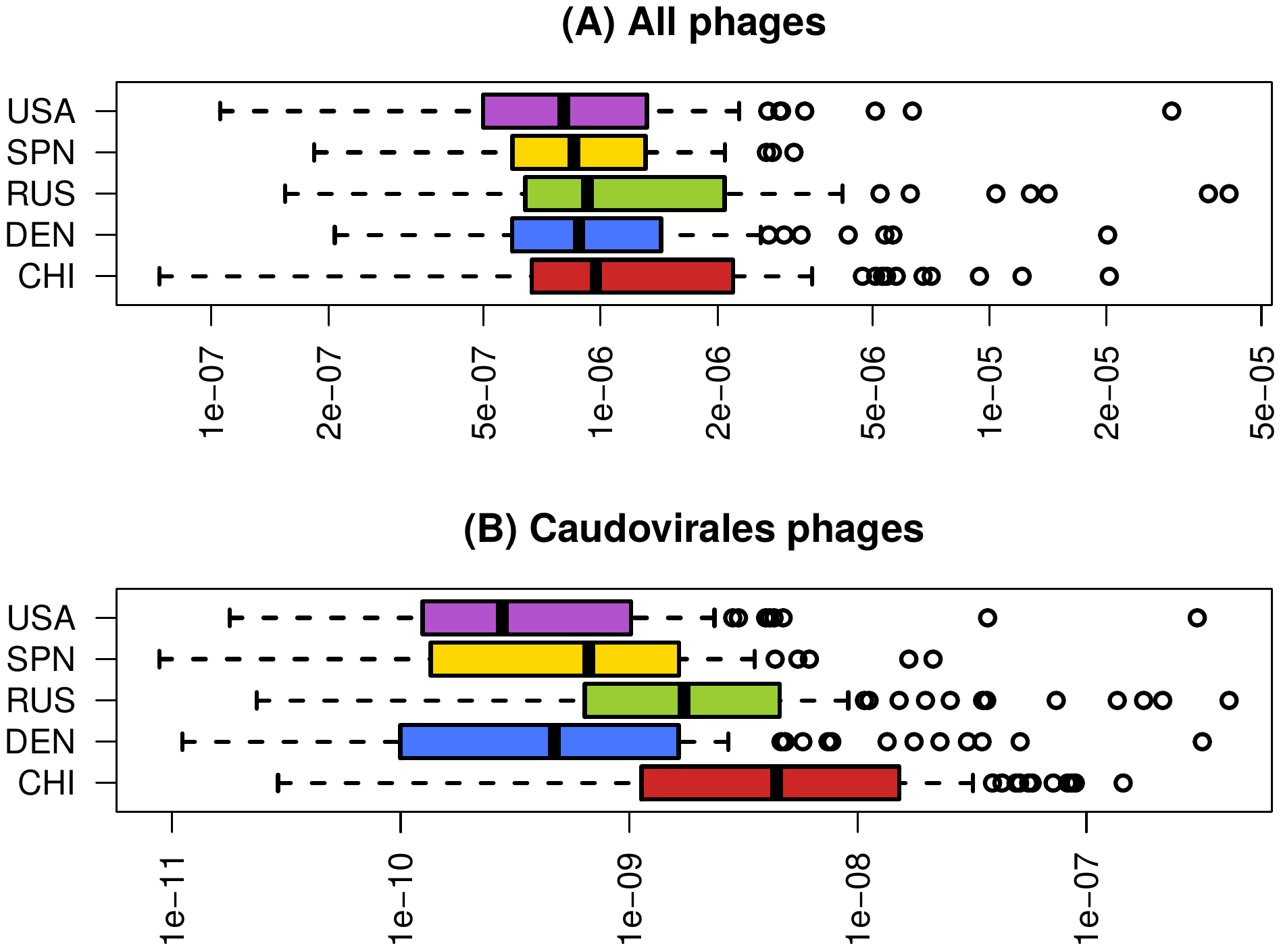

Supplement: S5 Fig — The abundance distribution in groups is shown with boxplots in logarithmic scale. A) Distribution of all phage terminases; B) Distribution of Caudovirales phages terminases only. (TIFF) [file pone.0176154.s010.tiff]

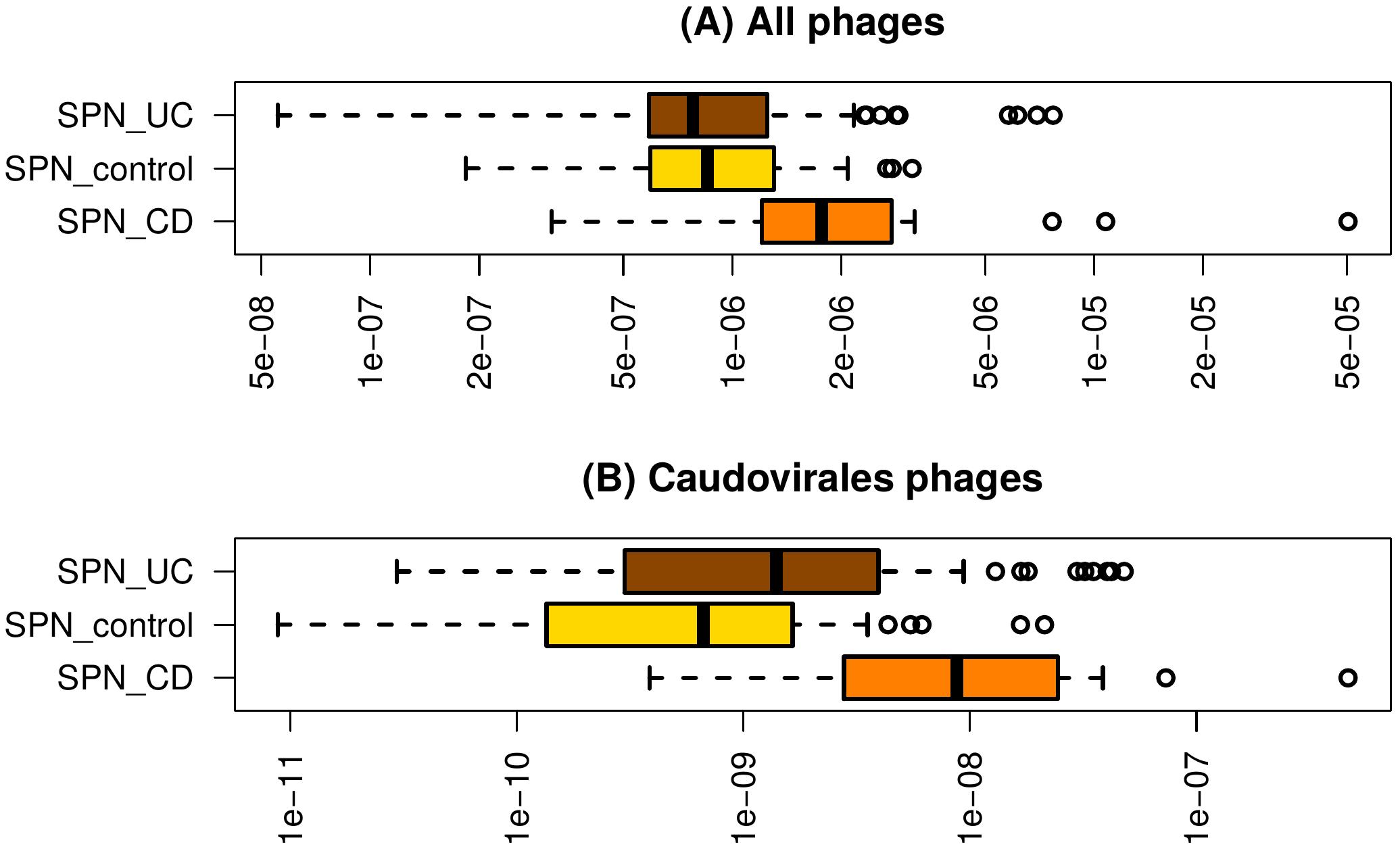

Supplement: S6 Fig — The abundance distribution in groups is shown with boxplots in logarithmic scale. A) Distribution of all phage terminases; B) Distribution of Caudovirales phages terminases only. (TIFF) [file pone.0176154.s011.tiff]

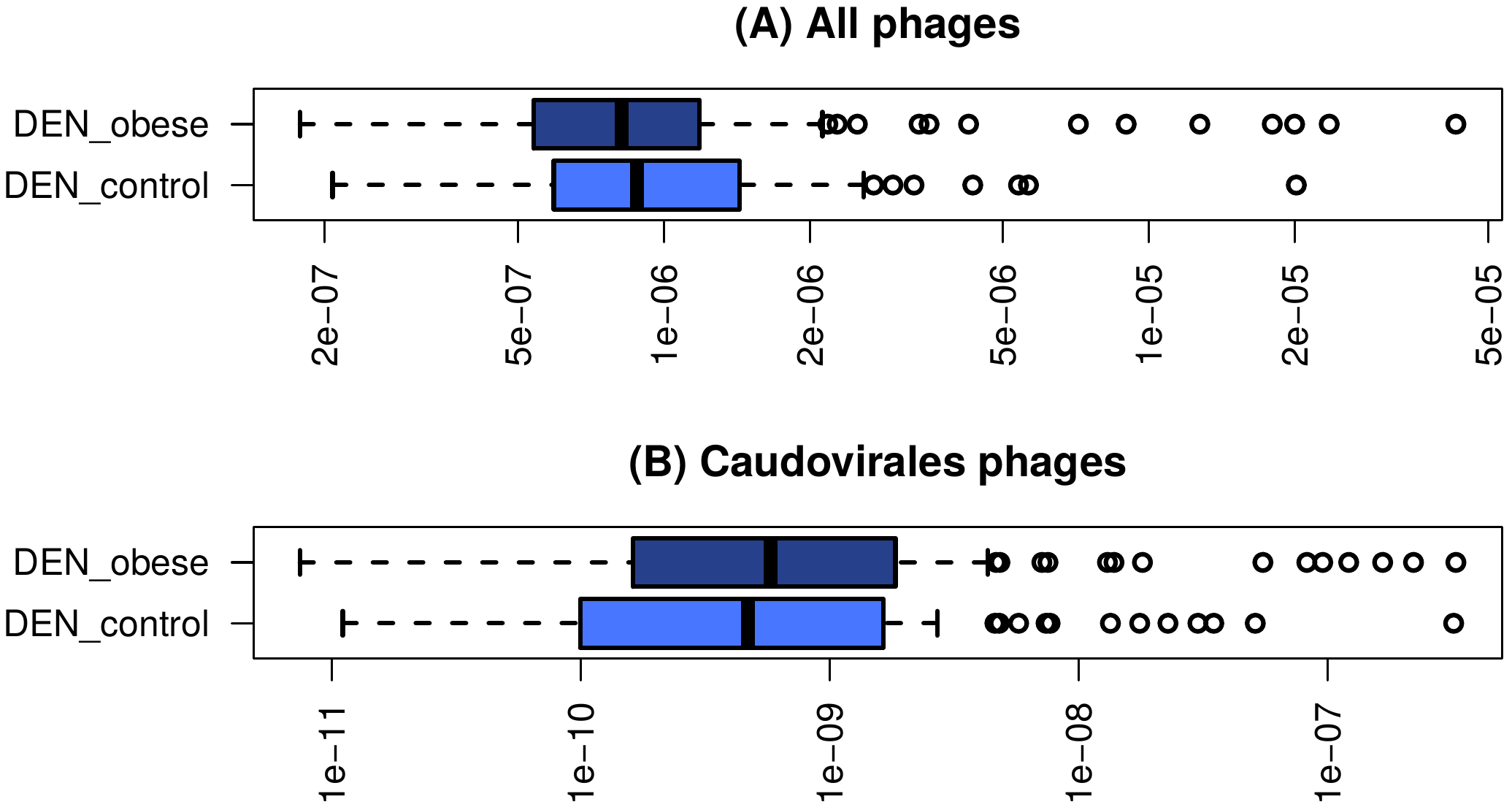

Supplement: S7 Fig — The abundance distribution in groups is shown with boxplots in logarithmic scale. A) Distribution of all phage terminases; B) Distribution of Caudovirales phages terminases only. (TIFF) [file pone.0176154.s012.tiff]

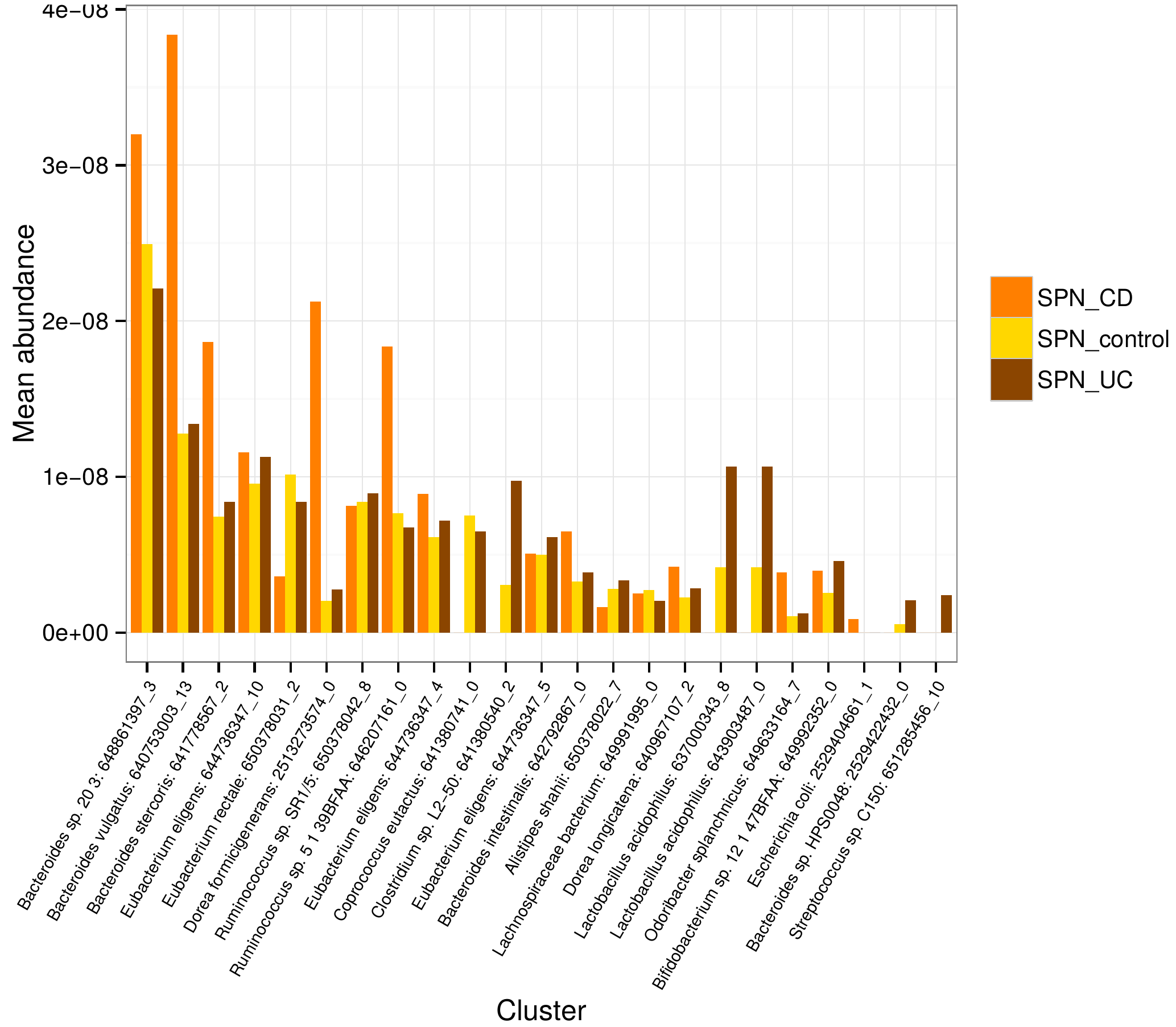

Supplement: S8 Fig — Cluster names along the X axis are indicated as in the original article [7]. For each cluster there are 3 colored bars for 3 sample groups, indicating mean abundance of RiPPs biosynthesis cluster in group. (TIFF) [file pone.0176154.s013.tiff]

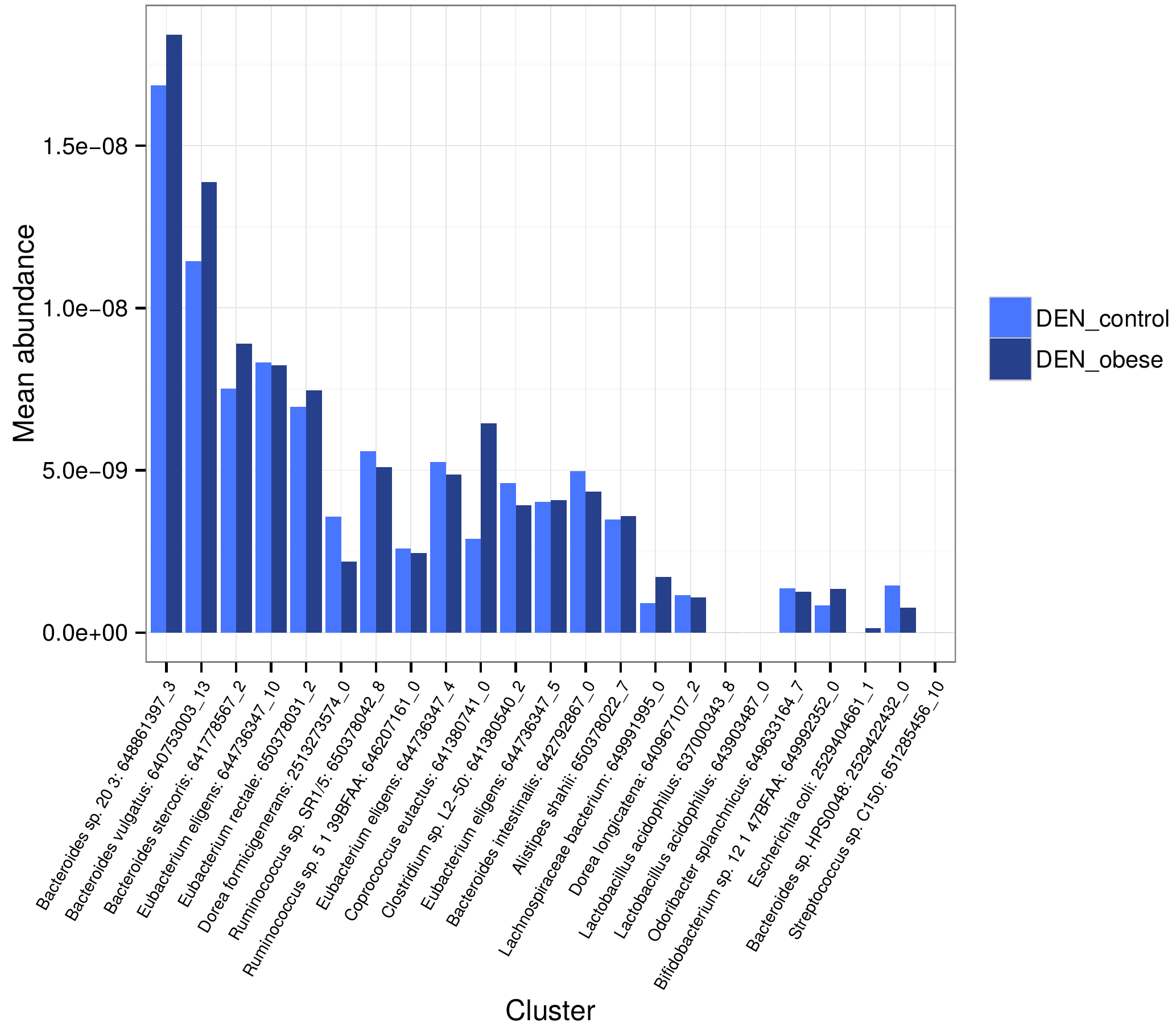

Supplement: S9 Fig — Cluster names along the X axis are indicated as in the original article [7]. For each cluster there are 2 colored bars for 2 sample groups, indicating mean abundance of RiPPs biosynthesis cluster in group. (TIFF) [file pone.0176154.s014.tiff]

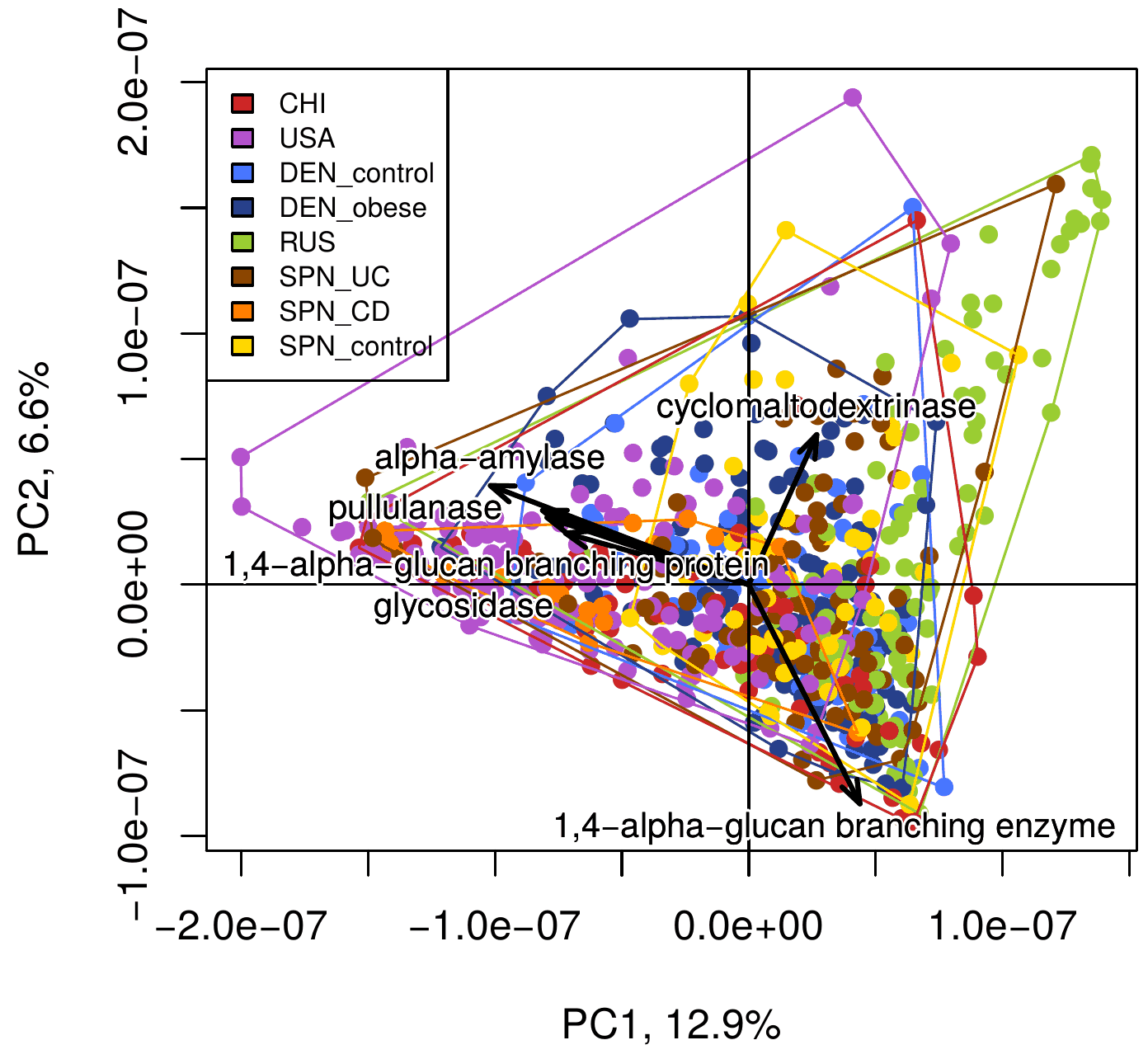

Supplement: S10 Fig — Arrows show the directions in which the levels of the 6 most abundant genes increase. Lines show convex hull for each cohort. (TIFF) [file pone.0176154.s015.tiff]

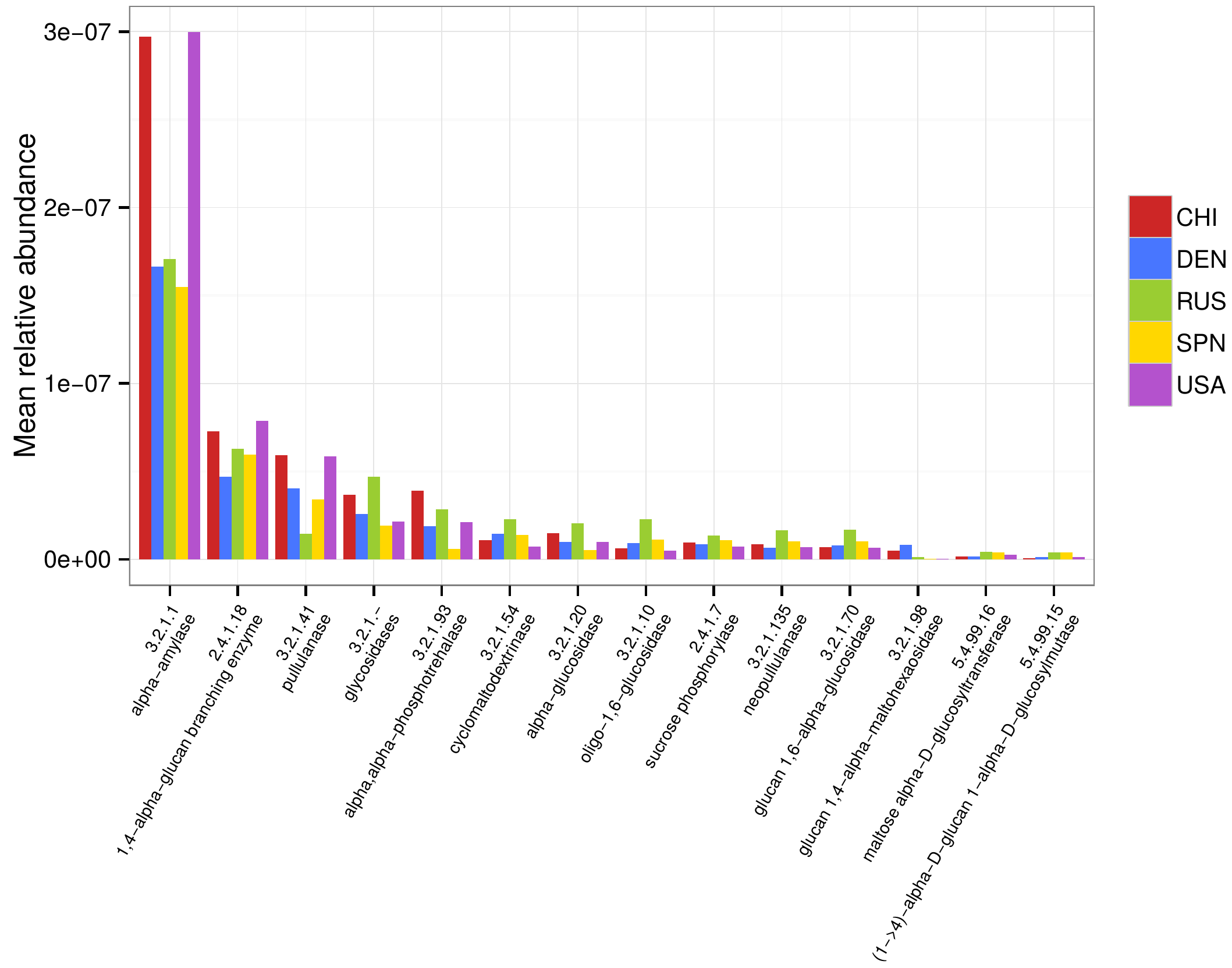

Supplement: S11 Fig — For each EC number, there are 5 bars for 5 sample groups indicating mean sum abundance of all genes with corresponding EC number in group. (TIFF) [file pone.0176154.s016.tiff]
